# Clinical features, imaging findings and molecular data of limb-girdle muscular dystrophies in a cohort of Chinese patients

**DOI:** 10.1186/s13023-023-02897-x

**Published:** 2023-11-16

**Authors:** Feng Lin, Kang Yang, Xin Lin, Ming Jin, Long Chen, Fu-ze Zheng, Liang-liang Qiu, Zhi-xian Ye, Hai-zhu Chen, Min-ting Lin, Ning Wang, Zhi-qiang Wang

**Affiliations:** 1 Department of Neurology and Institute of Neurology, The First Affiliated Hospital of Fujian Medical University, 20 Cha Zhong Road, Fuzhou, 350005 Fujian China; 2 Fujian Key Laboratory of Molecular Neurology, Fuzhou, 350005 China

**Keywords:** Muscular dystrophies, Clinical manifestations, Mutation, Respiratory insufficiency, Cardiac abnormalities

## Abstract

**Background:**

Limb-girdle muscular dystrophies (LGMDs) are a group of heterogeneous inherited diseases predominantly characterized by limb-girdle muscle weakness and dystrophic changes on histological analysis. The frequency of LGMD subtypes varies among regions in China and ethnic populations worldwide. Here, we analyzed the prevalence of LGMD subtypes, their corresponding clinical manifestations, and molecular data in a cohort of LGMD patients in Southeast China.

**Methods:**

A total of 81 consecutive patients with clinically suspected LGMDs from 62 unrelated families across Southeast China were recruited for targeted next-generation sequencing and whole-exome sequencing from July 2017 to February 2020.

**Results:**

Among 50 patients (41 families) with LGMDs, the most common subtypes were LGMD-R2/LGMD2B (36.6%) and LGMD-R1/LGMD2A (29.3%). Dystroglycanopathies (including LGMD-R9/LGMD2I, LGMD-R11/LGMD2K, LGMD-R14/LGMD2N and LGMD-R20/LGMD2U) were the most common childhood-onset subtypes and were found in 12.2% of the families. A total of 14.6% of the families had the LGMD-R7/LGMD2G subtype, and the mutation c.26_33dupAGGTGTCG in *TCAP* was the most frequent (83.3%). The only patient with the rare subtype LGMD-R18/LGMD2S had *TRAPPC11* mutations; had a later onset than those previously reported, and presented with proximal‒distal muscle weakness, walking aid dependency, fatty liver disease and diabetes at 33 years of age. A total of 22.0% of the patients had cardiac abnormalities, and one patient with *LMNA*-related muscular dystrophy/LGMD1B experienced sudden cardiac death at 37 years of age. A total of 15.4% of the patients had restrictive respiratory insufficiency. Muscle imaging in patients with LGMD-R1/LGMD2A and LGMD-R2/LGMD2B showed subtle differences, including more severe fatty infiltration of the posterior thigh muscles in those with LGMD-R1/LGMD2A and edema in the lower leg muscles in those with LGMD-R2/LGMD2B.

**Conclusion:**

We determined the prevalence of different LGMD subtypes in Southeast China, described the detailed clinical manifestations and distinct muscle MRI patterns of these LGMD subtypes and reported the frequent mutations and the cardiorespiratory involvement frequency in our cohort, all of which might facilitate the differential diagnosis of LGMDs, allowing more timely treatment and guiding future clinical trials.

**Supplementary Information:**

The online version contains supplementary material available at 10.1186/s13023-023-02897-x.

## Introduction

Limb-girdle muscular dystrophies (LGMDs) are a heterogeneous group of inherited disorders characterized by progressive weakness, wasting of proximal muscles, and dystrophic features on muscle biopsy. LGMDs are subdivided into autosomal-dominant LGMD1 and autosomal-recessive LGMD2. A new classification of five autosomal-dominant LGMDs (D1–D5) and twenty-four autosomal-recessive LGMDs (R1–R24) was proposed at the 229th European Neuromuscular Center (ENMC) workshop and to date, at least 34 reported genes have been associated with LGMDs [1].

The most common LGMDs are as follows: (a) Calpainopathy, also named LGMD-R1-calpain3-related, is one of the most prevalent forms of recessive LGMD, representing approximately 30–40% of LGMD cases with an estimated prevalence of 6.8–10.2 per million worldwide [2]. Disease onset usually occurs between 6 and 18 years but can range from 2 to 49 years of age. Approximately 80% of patients usually become wheelchair-bound between the second and fourth decades of life [3]. Calpainopathy is caused by a defect in the calpain-3 gene (*CAPN3*) located in the chromosomal region 15q15.1-q21.1. *CAPN3* encodes the muscle-specific calpain-3 protein that plays an important role in promoting the release of Ca^2+^ from skeletal muscle fibers, sarcomere remodeling, muscle contraction and NF-κB signaling [4, 5]. To date, more than 580 different pathogenic mutations have been reported, and these mutations are distributed along the entire coding region of the *CAPN3* gene. Two hotspot mutations, c.2120A > G and c.550del, were identified in Chinese and some European populations, respectively [6, 7]. Loss-of-function mutations of *CAPN3* inactivate its proteolytic function, playing an important role in the pathogenesis of calpainopathy, but the precise mechanism of calpainopathy has remained elusive. b) Autosomal recessive dysferlinopathy (R2) is one of the most commonly diagnosed LGMD subtypes in the world, with an estimated prevalence of 5.7–9.4 per million individuals. The clinical phenotypes of dysferlinopathy predominantly included Miyoshi myopathy, LGMD2B, and distal myopathy with anterior tibial onset (DMAT). The majority of patients with dysferlinopathy present progressive muscle weakness by the age of 30 years, but also later onset ranging to 60 years old has also been reported. Dysferlinopathy is caused by mutations in the *DYSF* gene located on chromosome 2p13.2, which spans a genomic region of approximately 233 kb and comprises 55 exons. More than 900 potentially deleterious *DYSF* variants have been reported, and hotspot mutations have been identified in some countries, such as c.2997G > T in Japanese patients and c.1375dup in Chinese patients [8, 9]. *DYSF* encodes the 237 kDa transmembrane protein dysferlin with functions associated with Ca^2+^ homeostasis, vesicle trafficking, sarcolemmal resealing and T‐tubule system shaping [10, 11]. Patients with *DYSF* mutations exhibit a deficiency of dysferlin protein in skeletal muscle membranes, and disruption of the aforementioned process contributes to the pathological mechanism.

(c) Sarcoglycanopathies (R3-R6) are the most severe form of LGMD, accounting for 10–25% of LGMDs. The prevalence of sarcoglycanopathies is variable in different ethnicities and geographic regions, ranging from 0.31/100,000 to 0.58/100,000 [2]. Most patients presented with progressive proximal muscle weakness with childhood onset, rapid progression and loss of ambulation in adolescence or early adulthood, although milder cases are still ambulant at age 60 years [12, 13]. Sarcoglycanopathies include four subtypes of LGMD (LGMDR3-R6), caused by recessive mutations in the *SGCA*, *SGCB*, *SGCG* and *SGCD* genes. Their four encoded corresponding proteins, α-sarcoglycan, β-sarcoglycan, γ-sarcoglycan and δ-sarcoglycan, are components of the dystrophin-glycoprotein complex (DGC) in the muscle sarcolemma. Mutations in these four sarcoglycan-related genes result in deficiency of the corresponding protein subunits, perturbing the heterotetramer complex closely linked to the dystrophin-associated protein complex in the cell membrane, which plays a critical protective role in membrane integrity and provides a scaffold for transmitting important signals [14]. (d) Several subtypes of LGMDs are dystroglycanopathies, primarily caused by mutations in eight genes, including *FKRP* (R9), *POMT1* (R11), *FKTN* (R13), *POMT2* (R14), *POMGnT1* (R15), *GMPPB* (R19), *ISPD* (R20), and *POMGNT2* (R24), involved in the glycosylation of the alpha-dystroglycan pathway [15]. Dystroglycan is a highly glycosylated adhesion receptor complex that is composed of alpha-dystroglycan and beta-dystroglycan subunits. Alpha-dystroglycan acts as a receptor that interacts with extracellular matrix partners, whereas beta-dystroglycan is a transmembrane protein that binds to dystrophin intracellularly and interacts with alpha-dystroglycan extracellularly. Alpha-dystroglycan and beta-dystroglycan interact noncovalently and provide a vital molecular link connecting the extracellular matrix to the internal machinery [16, 17]. Allelic mutations in dystroglycan-related genes encoding dystroglycan itself or glycosyltransferases and accessory proteins associated with the posttranslational modification of α-dystroglycan disrupt the O-glycosylation of α-DG and result in the loss of α-DG binding to its extracellular ligands, causing muscular dystrophy. Dystroglycanopathies have a wide spectrum of clinical phenotypes, ranging from severe congenital muscular dystrophy to a relatively milder form of LGMD [18, 19]. Among LGMD-related dystroglycanopathies, LGMD-R9-*FKRP*-related is the most frequent in North-European populations, with an estimated prevalence of 5.7–11.4 per million individuals [20, 21]. Most LGMD-R9 patients harbor at least one copy of the common mutation c.826C > A in the *FKRP* gene, and c.826C > A homozygotes manifest a milder phenotype than that of most other genotypes [22].

The mutational spectrum and prevalence of LGMD subtypes vary greatly among different geographical regions and ethnic populations worldwide. The LGMD-R1-calpain3-related and dysferlinopathy subtypes are the most common in the United States, India and Brazil [23–25], whereas LGMD-R1-calpain3-related and sarcoglycanopathies (LGMD-R3-5/LGMD2C-E) occur most frequently in the Netherlands. LGMD-R9-*FKRP*-related exhibits the highest prevalence in some European regions [26, 27]. To date, integrated data about LGMD epidemiology in the Chinese population are lacking, with only a few single-center studies on the LGMD subtypes in different geographic regions of China [28–30]. Moreover, the frequencies and spectrum of LGMD subtypes in these Chinese studies differed substantially. A definitive molecular diagnosis is important for patient management and participation in clinical trials, prognosis-based counseling and therapy selection.

The precise diagnostic approaches for LGMD subtypes depend on targeted protein immunohistochemistry (IHC) and molecular testing results. Considering the increasing numbers of LGMD subtypes and their corresponding clinical phenotypes, the absence of specific tests to determine the defective proteins in the most recently described subtypes and the time-consuming nature of conventional sequencing technologies for complex LGMD subtypes present a challenge. High-throughput genetic diagnostic technologies, including whole-exome sequencing (WES) and next-generation sequencing (NGS), are widely used to screen mutations in complex genetic diseases and have facilitated high diagnostic efficiency. In this study, we validated the use of targeted NGS and WES for mutation detection in a cohort of Chinese patients clinically suspected of having LGMDs at single center in Southeast China. To better estimate the frequencies and spectrum of LGMD subtypes in Chinese patients, we analyzed detailed clinical phenotypes and variant data in 81 patients from 62 unrelated families. This study aimed to confirm the existence of recurrent mutations in different LGMD subtypes in the Chinese population and to describe the clinical features of these LGMD subtypes. To date, this study is the first of an LGMD cohort from a single center specializing in neuromuscular diseases (NMDs) in Southeast China, and the results expand the clinical phenotypes and genetic profiles of LGMD in Chinese patients.

## Materials and methods

### Patients

This retrospective study included 81 consecutive patients from 62 unrelated families who were admitted to the First Affiliated Hospital of Fujian Medical University between July 2017 and February 2020 (Additional file 1: Table S1). The inclusion criteria were as follows: (a) age of onset > 2 years and a clinical phenotype including progressive proximal muscle weakness [31]; (b) dystrophic or myopathic changes in pathology when muscle biopsy specimens were available, including absent or a severe reduction in muscle-related proteins in immunohistochemical or western blotting analyses; and (c) facioscapulohumeral muscular dystrophy, Duchenne and Becker muscular dystrophy, myotonic muscular dystrophy, mitochondrial myopathy, lipid storage myopathy and glycogen storage myopathy were excluded. A detailed clinical history was obtained for all patients, including the age of onset, initial symptoms, disease duration, family pedigrees and neurological examination results. Serum creatine kinase (CK) activity and electromyography, muscle magnetic resonance imaging (MRI) and pulmonary function testing results were reviewed when available. Functional activity was evaluated using the Gardner–Medwin and Walton (GMW) scale [32]. Immunohistochemical analyses of muscle specimens were performed with primary antibodies against calpain-3, dysferlin, sarcoglycan and dystrophin. The protocols were approved by the ethics committee of The First Affiliated Hospital of Fujian Medical University and were conducted in accordance with the 1964 Declaration of Helsinki and its later amendments or comparable ethical standards. Informed consent was obtained from each participant.

### Muscle MRI

Muscle MRI of the lower limbs was performed with a 3.0-T whole-body scanner (Magnetom Verio; Siemens AG, Erlangen, Germany). T1-weighted, T2-weighted and fat-suppressed T2-weighted MRI series were acquired in both horizontal and coronal views. Fatty infiltration of the leg muscles was quantified by increased signal intensity as previously reported [33]. Fatty infiltration was scored as follows: 0 = normal appearance; 1 = mild involvement, slight hyperintense signal; 2 = moderate involvement, hyperintense signal in < 50% of the muscle; 3 = severe involvement, hyperintense signal in > 50% of the muscle and 4 = end stage, hyperintense signal in the entire muscle. Skeletal muscle edema was determined as high signal intensity on the fat-suppressed T2-weighted MR images and scored 0–5 as previously described [34]. The quality of the MRI scans was evaluated separately by two neuroradiologists blinded to the patients’ clinical features.

### Mutation detection and data analysis

Genomic DNA was collected from peripheral blood samples using the QIAmp DNA Blood Mini Kit (Qiagen, Hilden, Germany). Targeted NGS and WES analyses were conducted. Targeted NGS was performed using a panel (Agilent Technologies, Santa Clara, CA, USA) of inherited NMD-associated genes, as described previously [35]. WES was performed by Running Gene Inc. (Beijing, China) using the SureSelect Human All Exon Kit V6 (Agilent Technologies, Santa Clara, CA, USA). DNA samples were fragmented with the KAPA Library Preparation Kit (Illumina, Inc., USA) to build a DNA library. Then, the prepared libraries were sequenced using paired-end 150-nt reads on the Illumina HiSeq X10 platform (Illumina, San Diego, USA). The Burrows–Wheeler Aligner was applied to align the sequence data to the human reference genome (GRCh37/hg19). The Genome Analysis Toolkit (GATK) was used to call variants (including insertions, deletions and single-nucleotide variants (SNVs). Next, the annotation of the variants was performed by ANNOVAR. Variant frequency was filtered with a > 0.1% threshold according to public databases (the 1000 Genomes Project, gnomAD, ExAC and ESP databases). The sequence variants were interpreted according to the American College of Medical Genetics and Genomics (ACMG) standards and guidelines. Cosegregation analysis of the variants was performed in affected and unaffected family members. Variant analysis was performed by the detailed data interpretation process [36–39] (Additional file 2: Figure S1). Analysis of gene function was performed according to previously published literature and the OMIM database. The variants screened by targeted NGS and WES were further confirmed by Sanger sequencing. Sequence analysis was performed with reference sequences of *LMNA*(NM_170707.3), *CAPN3*(NM_000070.2), *DYSF*(NM_003494.3), *TCAP*(NM_003673.3), *FKRP*(NM_024301.4), *TTN*(NM_003319.4), *POMT1*(NM_007171.3), *POMT2*(NM_013382.5), *TRAPPC11*(NM_021942.5), and *ISPD*(NM_001101426.3) (Additional file 5).

### Statistical analysis

Descriptive statistics were used to evaluate variables (onset age and serum CK level). The onset ages, muscle scores and CK levels in different groups were determined by the Kruskal–Wallis test or Mann–Whitney test. The frequency of clinical symptoms in different subtypes was evaluated by Pearson’s chi-square or Fisher’s exact tests. Statistical significance was defined as a *P* values were < 0.05. Statistical analyses were performed by using GraphPad Prism 8.0.2.

## Results

**Fig. 1:**
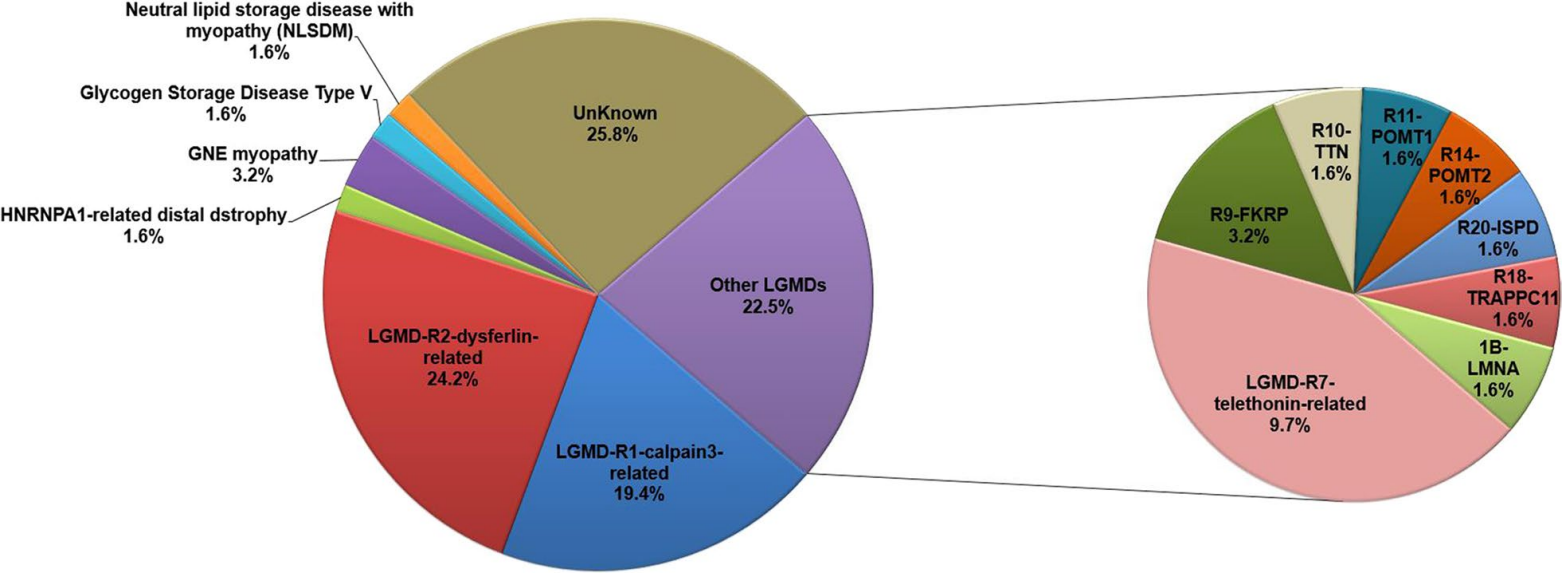
Frequencies of LGMD subtypes among patients with molecular diagnoses and the genetic spectrum of LGMD subtypes

We included 81 patients (62 probands and 19 of their affected relatives) from 62 unrelated families, 4 of whom were consanguineous. Among the 62 index patients, 46 probands were confirmed to carry one or more pathogenic variants in 14 different genes (Fig. 1), while no pathogenic variants associated with clinical and pathological phenotypes were found in the remaining 16 probands. Forty-one index patients and their 9 affected relatives were diagnosed with LGMDs clinically and genetically, including 40 patients with recessive LGMD and one with autosomal-dominant LGMD (Table 1). Twenty-two of the 40 recessive LGMD patients were confirmed to carry compound heterozygous mutations, and the remaining 18 carried homozygous mutations. Homozygous mutations were identified in the calpain 3 (*CAPN3*, 33.3%), dysferlin (*DYSF*, 33.3%) and titin-cap genes (*TCAP*, 100%). Sanger sequencing confirmed the presence of the mutations in all probands and confirmed cosegregation patterns in 25 pedigrees and their 72 members.

**Table 1:** Clinical and molecular data of patients with LGMDs

Subtype/Gene	Family	Age (y)/Gender	Onset age (y)	Motor function/Muscle involvement	CK level	Cardiac Dysfunction	Respiratory Involvement	Mutations
*LMNA*-related Muscular dystrophy/ LGMD1B(*LMNA,* NM_170707.3)	1	37/M	5	Ambulant neck flexors (+) proximal (+);distal (+)	1099	Cardiac arrhythmia LV dysfunction	–	Hetero c.746G > A
LGMD-R1/ LGMD2A (*CAPN3*, NM_000070.2)	2	25/M	16	Ambulant neck flexors (−) proximal (+);distal (+)	6598	Normal	Normal	Homo c.1621C > T
	3	48/M	28	Ambulant neck flexors (−) proximal (+);distal (−)	2178	–	–	Comp hetero c.433C > T c.1621C > T
	4(P1)	43/M	35	Ambulant neck flexors (−) proximal (+);distal (−)	3269	Tachycardia LV dysfunction	Normal	Comp hetero c.2050+ 1G > A c.2120A > G
	4(P2)	40/F	28	Ambulant neck flexors (−) proximal (+);distal (−)	1638	–	–	Comp hetero c.2050+ 1G > A c.2120A > G
	4(P3)	39/M	30	Ambulant neck flexors (+) proximal (+);distal (−)	1400	Normal	Normal	Comp hetero c.2050+ 1G > A c.2120A > G
	5	34/M	12	Walk aid dependency neck flexors (+) proximal (+);distal (+)	564	Normal	–	Comp hetero c.2305C > T c.1343G > A
	6	17/M	17	Ambulant neck flexors (−) proximal:upper limbs (−) lower limbs (+) distal (−)	7250	Normal	Normal	Comp hetero c.1720 T > G c.2306G > C
	7(P1)	41/M	20	Ambulant neck flexors (+) proximal (+);distal (−)	446	Sinus tachycardia LV dysfunction	–	Homo c.2263G > A
	7(P2)	38/F	13	Wheel−chair dependency neck flexors (+) proximal (+) distal (+)	1357	–	–	Homo c.2263G > A
	8	27/M	24	Ambulant neck flexors (−) proximal (+) distal lower limbs (+)	5241	Normal	–	Comp hetero c.2120A > G c.1451 T > C
	9	24/F	19	Ambulant neck flexors (−) proximal (+);distal (−)	3501	Normal	Normal	Comphetero c.1855C > T c.77C > G
	10	15/F	12	Ambulant neck flexors (+) proximal (+);distal (−)	1837	Normal	Normal	Homoc.2306G > C
	11	30/F	22	Ambulant neck flexors (−) proximal (+);distal (−)	–	–	–	Homoc.1817C > T
	12	47/M	27	Ambulant neck flexors (−) proximal (+);distal (−)	2123	–	–	Comp hetero c.2306G > C c.2120A > G
	13	20/M	10	Ambulant neck flexors (+) proximal (+);distal (−)	2144	Normal	Normal	Comp hetero c.1621C > T c.1693C > T
LGMD-R2/ dysferlinopathy (*DYSF*, NM_003494.3)	14(P1)	51/F	21	Wheel−chair dependency neck flexors (+) proximal (+);distal (+)	1142	Sinus tachycardia	Respiratory insufficiency	Homo c.863A > T
	14(P2)	60/F	32	Wheel−chair dependency neck flexors (+) proximal (+);distal (+)	1579	–	–	Homo c.863A > T
	15	20/M	19	Ambulant neck flexors (−) proximal: upper limbs (−) lower limbs (+) distal (−)	31,740	Normal	Normal	Comp hetero c.6217A > G c.5350C > T
	16	35/M	30	Ambulant neck flexors (+) proximal (+);distal (−)	5283	Normal	–	Comp hetero c.927C > G c.4700delT
	17	54/M	48	Ambulant neck flexors(−) proximal (+); distal (−)	3170	LV dysfunction	Normal	Comp hetero c.965 T > C c.1667 T > C
	18	24/M	18	Ambulant neck flexors (+) proximal (+) distal lower limbs (+)	10,530	Normal	–	Homo c.1667 T > C
	19(P1)	21/F	20	Ambulant neck flexors (+) proximal (+);distal (−)	6551	Normal	Normal	Comp hetero c.3102C > G c.5836C > T
	19(P2)	17/M	13	Ambulant neck flexors (−) proximal (+) distal lower limbs (+)	4474	Left ventricular high voltage	–	Comp hetero c.3102C > G c.5836C > T
	20	29/F	26	Ambulant neck flexors (+) proximal (+);distal (−)	19,611	Normal	Normal	Comphetero c.89-2A > G c.2810+ 1G > A
	21(P1)	33/F	23	Ambulant neck flexors (−) proximal: upper limbs (−) lower limbs (+);distal (−)	5407	–	–	Homo c.799_800delTT
	21(P2)	34/M	Not yet	Ambulant neck flexors (−) proximal (−);distal (−)	9045	Normal	Normal	Homo c.799_800delTT
	22	33/F	30	Ambulant neck flexors (−) proximal: upper limbs (+) lower limbs (−);distal (−)	3586	Normal	Normal	Comp hetero c.937+ 1G > A c.3113G > A
	23	37/M	17	Walk aid dependency neck flexors (+) proximal (+);distal (+)	1808	Normal	–	Homo c.5903G > A
	24	27/M	26	Ambulant neck flexors (−) proximal: upper limbs (−) lower limbs (+);distal (−)	4358	Normal	–	Comp hetero c.5694dupT c.937+ 1G > A
	25	29/F	25	Ambulant neck flexors (−) proximal: upper limbs (−) lower limbs (+);distal (−)	12,140	Normal	Normal	Comp hetero c.4325delG c.5947-1G > A
	26	40/M	28	Ambulant neck flexors (−) proximal:upper limbs (−) lower limbs (+) distal lower limbs (+)	3284	Normal	Respiratory insufficiency	Comp hetero c.863A > T 55 exon deletion
	27	23/M	20	Ambulant neck flexors(−) proximal(−) distal lower limbs(+)	9255	Normal	Normal	Comp hetero c.712A > T c.4200delC
	28	33/F	18	Ambulant neck flexors (−) proximal: upper limbs (−) lower limbs (+) distal lower limbs (+)	2987	Normal	Normal	Homo c.1644delA
LGMD-R7/ LGMD2G (*TCAP*, NM_003673.3)	29	32/M	22	Ambulant neck flexors (+) proximal (+);distal (−)	974	–	Normal	Homo c.26_33dupAGGTGTCG
	30	25/F	22	Ambulant neck flexors (−) proximal: upper limbs (−) lower limbs (+) distal lower limbs (+)	678	Normal	Normal	Homo c.26_33dupAGGTGTCG
	31	33/F	15	Ambulant neck flexors (−) proximal: upper limbs (−) lower limbs (+) distal lower limbs (+)	872	Normal	Normal	Homo c.26_33dupAGGTGTCG
	32	33/M	16	Ambulant neck flexors (−) proximal: upper limbs (−) lower limbs (+) distal lower limbs (+)	2361	–	–	Homo c.26_33dupAGGTGTCG
	33	48/M	17	Ambulant neck flexors (+) proximal (+) distal lower limbs (+)	408	Normal	–	Homo c.110+ 5G > A
	34(P1)	41/F	31	Ambulant neck flexors (+) proximal (+) distal lower limbs (+)	1357	Normal	Normal	Homo c.26_33dupAGGTGTCG
	34(P2)	37/F	35	Ambulant neck flexors (−) proximal: upper limbs (−) lower limbs (+) distal lower limbs (+)	823	Normal	–	Homo c.26_33dupAGGTGTCG
LGMD-R9/ LGMD2I (*FKRP*, NM_024301.4)	35	29/F	10	Walk aid dependency neck flexors (+) proximal (+);distal (+)	1325	Cardiomyopathy	Respiratory insufficiency	Comp hetero c.948delC c.545A > G
	36	8/F	6	Ambulant neck flexors (+) proximal (+);distal (−)	6300	Normal	Normal	Comp hetero c.545A > G c.206_208delCCT
LGMD-R10/ LGMD2J (*TTN*, NM_003319.4)	37(P1)	23/M	8	Ambulant neck flexors (−) proximal: upper limbs (−) lower limbs (+);distal (−)	1025	Normal	–	Homo c.7509 T > C
	37(P2)	25/F	11	Ambulant neck flexors (−) proximal (+) distal lower limbs (+)	875	Normal	–	Homo c.7509 T > C
LGMD-R11/ LGMD2K (*POMT1*, NM_007171.3)	38(P1)	15/M	5	Ambulant neck flexors (−) proximal (+);distal (−)	1895	Early repolarization syndrome	–	Comp hetero c.2210_2221del c.2164G > A
	38(P2)	25/F	10	Ambulant neck flexors (−) proximal (+);distal (−)	1038	Normal	–	Comp hetero c.2210_2221del c.2164G > A
LGMD-R14/ LGMD2N (*POMT2*, NM_013382.5)	39	10/F	4	Ambulant neck flexors (+) proximal (+);distal (−)	12,671	Normal	–	Comp hetero c.511C > T c.365G > T
LGMD-R18/ LGMD2S (*TRAPPC11*, NM_021942.5)	40	33/F	19	Walk aid dependency neck flexors (+) proximal (+);distal (+)	420	Normal	Respiratory insufficiency	Homo c.2938G > A
LGMD-R20/ LGMD2U (*ISPD*, NM_001101426.3)	41	24/M	4	Wheel−chair dependency neck flexors (+) proximal (+);distal (+)	2807	Sinus tachycardia	Normal	Homo c.1114_1116del

LGMD, limb-girdle muscular dystrophy; P, patient; M, male; F, female; y, years; CK, creatine kinase; proximal, proximal limbs; distal, distal limbs; (+), involved; (−), not involved;–, not available

### Mutational spectrum of LGMDs in our cohort

Among the LGMD patients in our cohort, 18 patients from 15 unrelated families carried *DYSF* mutations, 15 patients from 12 different families carried *CAPN3* mutations, 7 patients from 6 families carried *TCAP* mutations, two patients carried fukutin-related protein (*FKRP*) mutations, two siblings from one family carried titin (*TTN*) mutations, two siblings carried protein-O-mannosyltransferase 1 (*POMT1*) mutations, one patient carried lamin A/C (*LMNA*) mutations, one patient carried protein-O-mannosyltransferase 2 (*POMT2*) mutations, one patient carried trafficking protein particle complex 11 (*TRAPPC11*) mutations, and one patient carried isoprenoid synthase domain-containing (*ISPD*) mutations (Table 1). The disease-causing mutations identified in index patients were distributed as follows: missense mutations, 50.6%; frameshift mutations, 22.2%; nonsense mutations, 11.1%; splice mutations, 9.9%; deletion mutations, 4.9%; and exonic rearrangements, 1.2%.

LGMD-R2-dysferlin-related was the most common subtype of LGMD (15/41 index patients, 36.6%) in our cohort. LGMD-R1-calpain3-related was the second most common LGMD subtype, found in 29.3% of the index patients. LGMD R7 telethonin-related was found in 14.6% (6/41) of index patients, and dystroglycanopathies (including mutations in *FKRP*, *POMT1*, *POMT2* and *ISPD*) were found in 12.2% (5/41) of index patients. The regional relative frequencies of the different subtypes of LGMD in China are shown in Table 2. Thus far, four cohorts of LGMD subtypes with different geographical distributions have been comprehensively analyzed in China, including the cohort in our study. Dysferlinopathy was the most common subtype of LGMD in the four cohorts, except in patients from Northeast China. Sarcoglycanopathies and dysferlinopathy are equally common in southern China, whereas in Taiwan, the most common diagnoses of LGMD subtypes are dystroglycanopathies and dysferlinopathy.

**Table 2:** Regional relative frequencies and clinical data of LGMD subtypes in mainland China and Taiwan

Item	Our study	Meng Yu et al	Qi Zhang et al	Liang Wang et al	Wen-Chen et al
Geographical distribution	Southeast China	North China	Northeast China	South China	Taiwanese
Confirmed LGMD Family	41	95	26	24	26
Number of cases	50	105	26	30	40
Males: Females	27:23	58:47	20:6	12:18	21:19
Age at onset (years)	19.7 ± 9.3	19.1 ± 11.5	31.6 ± 14.0	12.0 ± 7.9	15.8 ± 11.8
Median age of onset years (range)	19 (4–48)	17 (2–47)	28.5 (15–71)	13 (1.5–29)	14 (2–50)
Cardiac dysfunction	22.0% (9/41)	–	–	33.3% (3/9)	27.0% (10/37)
Restrictive pulmonary defect	15.4% (4/26)	–	–	–	47.4% (18/38)
Most Common subtype	Dysferlinopathy	Dysferlinopathy	Calpainopathy	Dysferlinopathy Sarcoglycanopathies	Dysferlinopathy Dystroglycanopathies
Calpainopathy	29.30%	24.80%	46.20%	16.70%	11.50%
Dysferlinopathy	36.60%	49.50%	38.40%	29.20%	23.10%
Sarcoglycanopathies	Not found	9.50%	7.70%	29.20%	15.40%
LGMD-R7/LGMD2G	14.60%	Not found	Not found	Not found	11.50%
Dystroglycanopathies	12.20%	3.80%	Not found	8.30%	23.10%
LGMD-R9/LGMD2I	4.90%	2.90%	Not found	8.30%	19.20%

LGMD,limb-girdlemusculardystrophy;-,notavailable

**Fig. 2:**
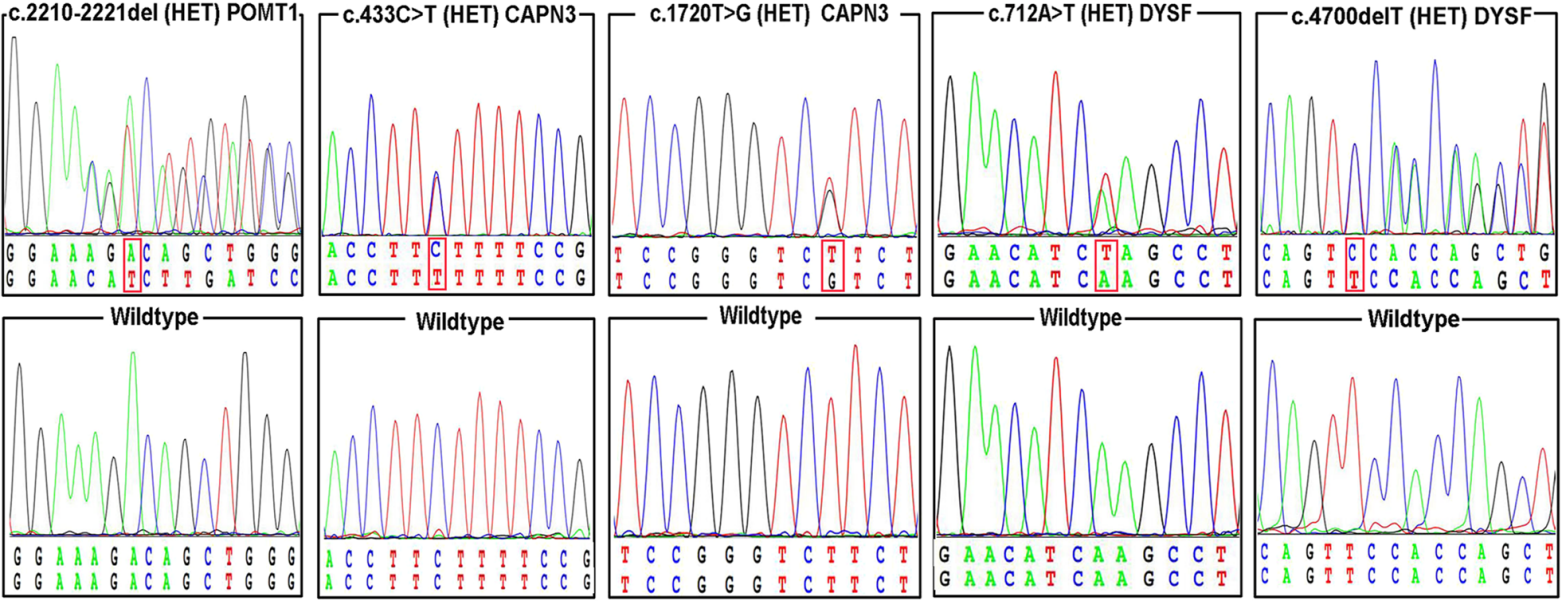
Identification of five novel mutations in our cohort. Sanger sequencing of the *POMT1*, *CAPN3* and *DYSF* mutations in the five patients and the corresponding wild-type subjects. Mutations are marked by red boxes

Several frequent mutations were observed in LGMD-R1-calpain3-related and LGMD-R7-telethonin-related patients. A high percentage (91.7%) of missense mutations was identified in the *CAPN3* gene in the analysis of the allele frequencies of pathogenetic mutations in LGMD-R1-calpain3-related. A total of fourteen different mutations were found in the *CAPN3* gene, and 85.7% of them were missense mutations. The missense mutations c.2306G > C(p.Arg769Pro), c.2120A > G(p.Asp707Gly) and c.1621C > T(p.Arg541Trp) were identified in three patients. Notably, the homozygous mutation c.26_33dupAGGTGTCG(p.Glu12Argfs*20) was detected in all patients with LGMD-R7-telethonin-related, except for one who carried homozygous c.110 + 5G > A mutations inherited from their consanguineous father and mother. In total, five novel mutations were detected: c.2210**_**2221del(p.Lys737_Asp741delinsN) in *POMT1*, c.433C > T(p.Leu145Phe) and c.1720 T > G(p.Arg574Val) in *CAPN3*, and c.4700delT(p.Phe1567Serfs*41) and c.712A > T(p.Lys238*) in *DYSF* (Fig. 2). None of the novel mutations were found in the 100 normal controls.

### Clinical overview of genetically diagnosed LGMDs

#### Clinical characteristics

The mean age at the initial onset of symptoms was 19.7 ± 9.3 years (range 4–48 years), while 9 patients had an age of onset before 11 years. The subgroup of patients with dystroglycanopathies had the youngest age of onset; all of them experienced muscle weakness before 11 years (between 4 and 10 years). Most of the patients with LGMD-R1-calpain3-related, dysferlinopathy and LGMD-R7-telethonin-related exhibited muscle weakness after the age of 15 years, and 8/15 (53.3%) patients with LGMD-R1-calpain3-related, 12/18 (66.7%) patients with dysferlinopathy and 4/7 (57.1%) patients with LGMD-R7-telethonin-related had an age of onset after 19 years; the mean ages of onset were 20.9 ± 7.6 years for patients with LGMD-R1-calpain3-related, 24.4 ± 8.1 years for patients with dysferlinopathy and 22.6 ± 7.7 years for patients with LGMD-R7-telethonin-related (*P* > *0.05*). Except for one patient with asymptomatic hyperCKemia, all of the patients had different degrees of proximal lower limb weakness, and 14 of the patients had no involvement of the proximal upper limbs, eight of whom had dysferlinopathy, one had LGMD-R1-calpain3-related, four had LGMD-R7-telethonin-related, and one had LGMD-R10-*TTN*-related. Twenty-eight patients had no involvement of the neck flexors, which were often affected along with the upper limbs.

Four patients had a loss of ambulation and developed wheelchair dependence, two of whom had dysferlinopathy, with a mean age of 56 years; one had LGMD-R1-calpain3-related and was 38 years old; and one had LGMD-R20-*ISPD*-related and was 21 years old. Four patients exhibited walking aid dependency at an average age of 33.3 years (one patient each with LGMD-R1-calpain3-related, dysferlinopathy, LGMD-R9-*FKRP*-related and LGMD-R18-*TRAPPC11*-related). Thirteen patients were able to walk independently but with obvious gait abnormalities and had difficulty jumping, climbing and standing up from squatting (LGMD-R1-calpain3-related: seven patients; dysferlinopathy: three patients; LGMD-R9-*FKRP*-related: one patient; LGMD-R14-*POMT2*-related: one patient; and *LMNA*-related muscular dystrophy: one patient). Relatively common clinical features in some subtypes of LGMD were observed; winged scapula occurred in 40% of individuals with LGMD-R1-calpain3-related, foot drops occurred in 33.3% of individuals with dysferlinopathy, pseudohypertrophy occurred in 66.7% of individuals with dystroglycanopathies, and joint contractures of the ankles were observed in three patients (one with LGMD-R7-telethonin-related, one with dysferlinopathy and one with LMNA-related muscular dystrophy).

#### Phenotype–genotype correlation

Of the 15 patients with LGMD-R1-calpain3-related, 11 harbored 2 missense mutations, and 4 harbored one missense mutation and one null (nonsense, frameshift, or splicing) mutation. The patients with two missense mutations had an earlier age at symptom onset (18.3 ± 6.3 years, range 10 to 28 years) than patients with one missense/null mutation (28.0 ± 6.7 years, range 19–35 years) (*P* < *0.05*). However, there was no significant difference in the GMW grade between patients with two missense mutations and those with one missense/null mutation (mean score 4.7 vs. 4.3, *P* > *0.05*).

Among the 18 patients with dysferlinopathy, 10 had two null (nonsense, frameshift, or splicing) mutations (Group 1), and 8 had at least one missense mutation (Group 2, including 4 with two missense mutations and 4 with one missense mutation and one null mutation) (Table 3). Patients in Group 1 had an earlier age at onset (median age 21.3 years) than those in Group 2 (median age 27.8 years), but this difference was not significant (*P* > *0.05*). In Group 1, members of one family (F21) showed marked phenotypic variability. One patient (F21, P1) experienced difficulty running at onset, presented with lower limb weakness at 23 years, and could not independently stand up from a squatting position after 6 years of follow-up. Her affected brother (F21, P2) showed asymptomatic hyperCKemia on neuromuscular examination at age 34 years. There were no significant differences between Group 1 and Group 2 in the clinical severity of the disease and laboratory findings (*P* > *0.05*).

**Table 3:** Clinical characteristics of patients with two null mutations and at least one missense mutation in *DYSF* gene

Clinical features	Group 1: two null mutations	Group 2: at least one missense mutations
Patients of numbers	10	8
Male: Female	5:5	5:3
Age at onset (years)	21.3 ± 5.2	27.8 ± 9.7
Mean age at examination (years)	28.6 ± 6.8	38.6 ± 15.0
Mean disease duration (years)	5.8 ± 6.0	10.9 ± 11.8
Clinical severity		
No upper limbs involvement (*n*)	3	5
Gardner-Medwin and Walton scale	3.3 ± 2.1	3.5 ± 2.9
Laboratory findings		
Mean CK level	7553.1 ± 5330.1	7423.7 ± 10,239.4
Cardiorespiratory involvement (*n*)	1	2

#### CK levels and cardiopulmonary function

**Fig. 3:**
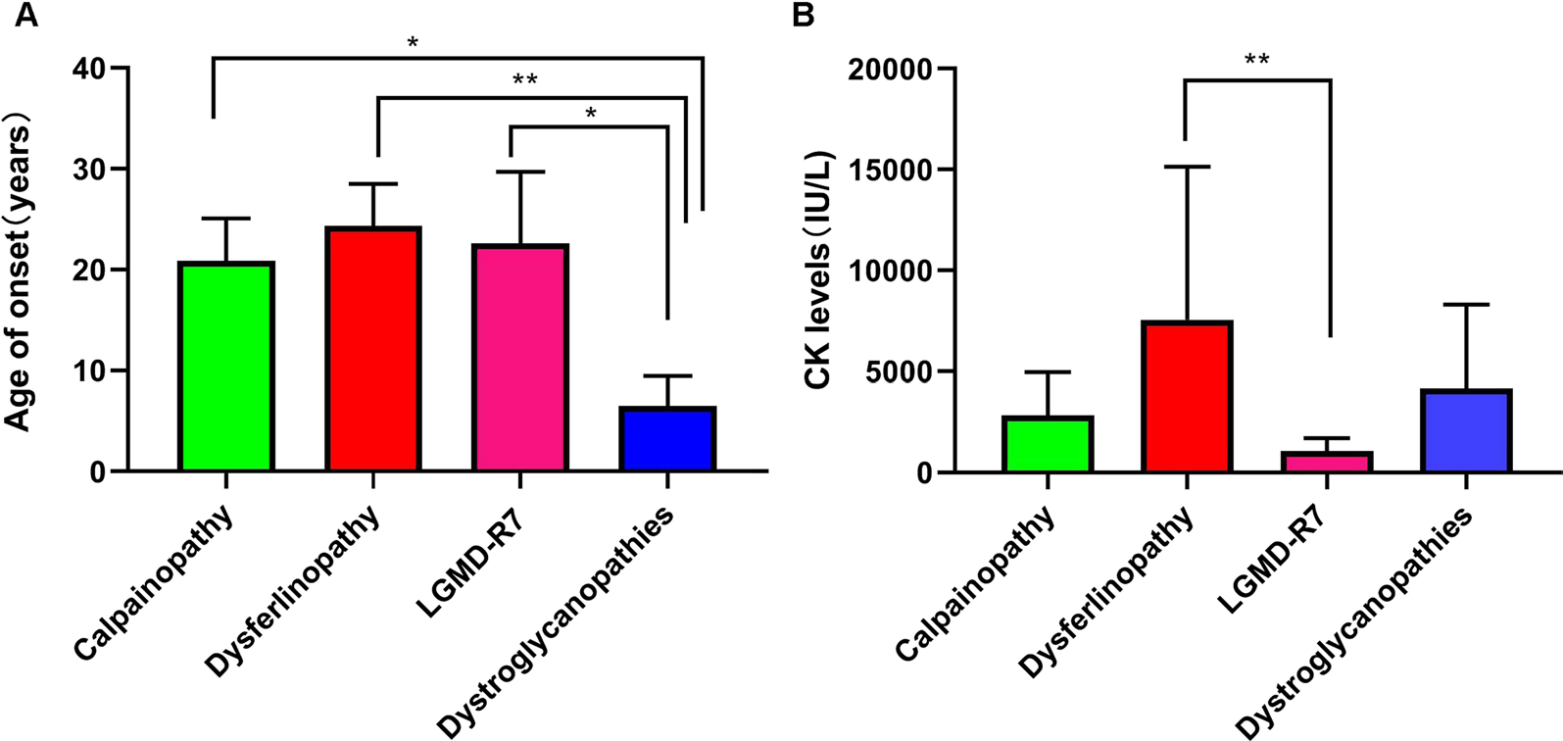
Age at onset and creatine kinase levels of common LGMD subtypes. **A** Age at onset of calpainopathy, dysferlinopathy, LGMD-R7-telethonin-related and dystroglycanopathies (LGMD-R9-*FKRP*-related, LGMD-R11-*POMT1*-related, LGMD-R14-*POMT2*-related and LGMD-R20-*ISPD*-related). **B** Creatine kinase levels in patients with calpainopathy, dysferlinopathy, LGMD-R7-telethonin-related and dystroglycanopathies; *P < 0.05, **P < 0.001

The median CK level was 2178 IU/L (range, 408–31740). CK levels were mildly to markedly increased in all patients. There was a significant difference in CK levels between individuals with dysferlinopathy and those with LGMD-R7-telethonin-related (*P* < *0.005*) (Fig. 3). Furthermore, the CK levels of all patients with LGMD-R7-telethonin-related were less than tenfold of the upper limit of normal (ULN). Among nineteen patients whose CK levels were more than tenfold higher and 50-fold lower than the ULN, twelve had dysferlinopathy, four had LGMD-R1-calpain3-related and two had dystroglycanopathies (one had LGMD-R9-*FKRP*-related and one had LGMD-R20-*ISPD*-related) *(P* > *0.05*). The serum CK levels of four patients were more than 50-fold over the ULN, three of whom had dysferlinopathy and one of whom had LGMD-R14-*POMT2*-related.

In the cardiac evaluations, none of the patients showed malignant arrhythmia or heart failure by electrocardiography (ECG), but one patient with LMNA-related muscular dystrophy/LGMD 1B suddenly died from cardiac disease. ECG showed cardiac arrhythmia in two patients with LGMD-R1-calpain3-related (average age of 42 years), and cardiac function evaluation revealed decreased diastolic function of the left ventricle (LV). Cardiac abnormalities were observed in three patients with LGMD-R2-dysferlin-related, including one with decreased diastolic function of the LV, one with high voltage in the LV and one with bradycardia. One patient with LGMD-R11-*POMT1*-related showed early repolarization syndrome, but the echocardiography results were normal. Another patient with LGMD-R9-*FKRP*-related showed a right bundle branch block, and cardiac magnetic resonance (CMR) indicated a slight enlargement and myocardial fatty infiltration of the LV. One patient with LMNA-related muscular dystrophy/LGMD 1B showed atrial flutter and intraventricular block, and CMR revealed an enlargement of the left atrium and right heart and decreased systolic and diastolic function of the LV; he experienced sudden cardiac death at age 37 years.

Twenty-six patients underwent a pulmonary function test. Four patients showed restrictive respiratory involvement, as indicated by the forced vital capacity (FVC), and the predicted FVC was 80%. The predicted FVC was 43.2% for one patient with LGMD-R9-*FKRP*-related at 29 years and 36.9% for one patient with LGMD-R18-*TRAPPC11*-related at 33 years. The predicted FVC was 62.4% and 71.2% for two patients with dysferlinopathy at 51 and 40 years old, respectively. One of the patients with dysferlinopathy used a wheelchair, and another two had a relatively severe phenotype with difficulty climbing stairs without aid, involving the proximal limbs for more than 10 years. In these patients, the reduction in the forced expiratory volume in 1 s (FEV1) was associated with the reduction in the FVC, with a preserved FEV1/FVC ratio similar to normal. The pCO_2_ level was mildly elevated by more than 45 mmHg in one patient with LGMD-R9-*FKRP*-related, and the pO_2_ was normal in all patients at rest. None of the patients used nocturnal noninvasive ventilation.

#### Muscle imaging

**Fig. 4:**
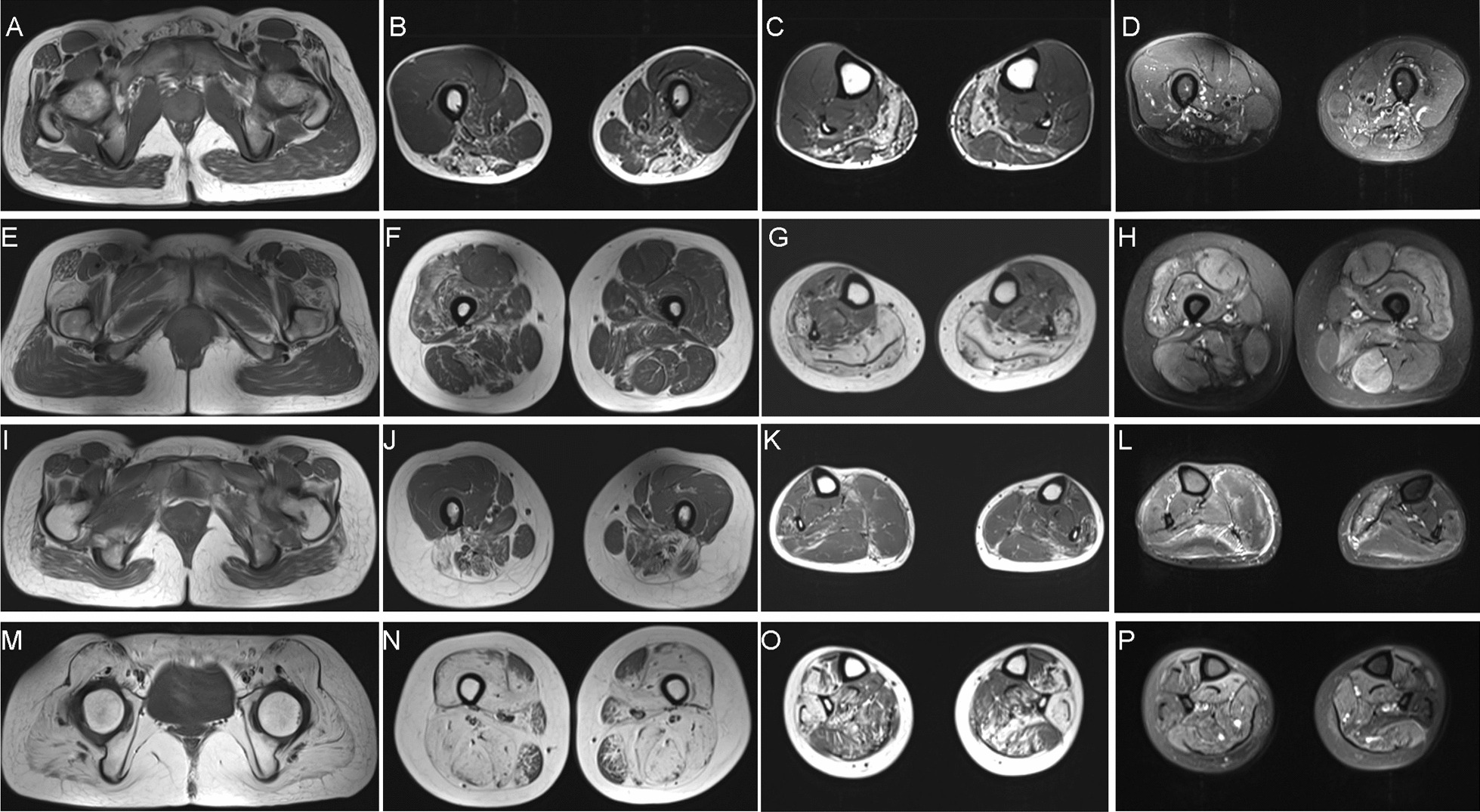
Representative T1-weighted images (**A**–**C**, **E**–**G**, **I**–**K**, **M**–**O**) and fat-suppressed T2-weighted MRI images (**D**, **H**, **L**, **P**) of LGMD-R1-calpain3-related, dysferlinopathy, LGMD-R7-telethonin-related, and LGMD-R9-*FKRP*-related (**A**–**D**). Muscle MRI of LGMD-R1-calpain3-related revealed severe involvement of the AM and posterior compartment muscles (including the SM, ST and BF at the thigh level and the GC and SO at the calf level), GR hypertrophy and a normal appearance of the RF, QF and S at the thigh level and of the TA, TP and EDL at the calf level. **E**–**H** Muscle MRI of a patient with dysferlinopathy indicated that the posterior and anterior compartments of the thigh muscles were equally involved and that fatty infiltration of the GC, SO and PL was severe. Compared with LGMD-R1-calpain3-related, the posterior compartment of the thigh muscles was less affected, but edema of the QF and ST was predominantly observed in dysferlinopathy. **I**–**L** LGMD-R7-telethonin-related, showed severe involvement of the GMa, hamstrings, BL and AM, but the QF was relatively preserved; marked and asymmetric edema was present in the GC, SO and TA. **M–P** In LGMD-R9-*FKRP*-related, the anterior and posterior muscles in the thigh were involved; however, the S, GR and RF were relatively preserved. At the calf level, the involvement of the PL and EDL was more severe than that of the GC and SO, whereas the TA was relatively spared. Edema of the lower legs showed the opposite pattern, in which the TA, GC, SO, TP and FDL were involved, but the PL and EDL were unaffected. *AM*: adductor magnus; *BF*: biceps femoris; *EDL*: extensor digitorum longus; *FDL*: flexor digitorum longus; *GC*: gastrocnemius; *GMa*: gluteus maximus; *GR*: gracilis; *PL*: peroneus longus; *RF*: rectus femoris; *QF*: quadriceps femoris; *S*: sartorius; *SO*: soleus; *ST*: semitendinosus; *SM*: semimembranosus; *TA*: tibialis anterior; *TP*: tibialis posterior)

Muscle MR imaging was performed on 28 patients (9 with LGMD-R1-calpain3-related, 13 with LGMD-R2-dysferlin-related, 3 with LGMD-R7-telethonin-related, 1 with LGMD-R9-*FKRP*-related, 1 with LGMD-R11-*POMT1*-related and 1 with LGMD-R18-*TRAPPC11*-related). Obvious fatty infiltration was observed in the lower legs of all patients, except for the patient with LGMD-R11-*POMT1*-related. In comparisons of the degree of fatty infiltration, patients with LGMD-R1-calpain3-related had more severe posterior thigh muscle fatty infiltration than those with LGMD-R2-dysferlin-related, but only adductor longus fatty infiltration significantly differed between the subtypes (*P* < *0.05*) (Additional File 3: Figure S2). One LGMD-R1-calpain3-related patient demonstrated a peculiar pattern: severe atrophy and fatty infiltration of the hamstrings and adductor magnus, gracilis hypertrophy and a normal appearance of the rectus femoris, quadriceps and sartorius (Fig. 4). In comparisons of the degree of edema, individuals with LGMD-R2-dysferlin-related showed more severe edema in the lower leg muscles than individuals with LGMD-R1-calpain3-related (Additional file 4: Figure S3).

In patients with LGMD-R7-telethonin-related, we observed that the hamstrings and gluteus muscles were involved earlier than the vastus lateralis and vastus intermedius anterior muscles. Edema of the lower leg muscles, including the gastrocnemius, soleus and tibialis anterior muscles, was obvious and asymmetric in individuals with LGMD-R7-telethonin-related. In the patient with LGMD-R9-*FKRP*-related, all of the thigh muscles exhibited severe fatty infiltration except for the sartorius, gracilis and rectus femoris, which were relatively preserved. The lower leg involvement in the patient with LGMD-R9-*FKRP*-related was peculiar. The peroneus longus muscle and extensor digitorum longus were more severely involved than the posterior compartment muscles, including the gastrocnemius, whereas the tibialis anterior muscle was relatively spared. However, edema of the tibialis muscles and the posterior compartment were observed, which revealed an opposite feature in the involved muscles. The thigh muscle involvement in the proband with LGMD-R18-*TRAPPC11*-related was severely affected at the late stage, revealing the fatty infiltration of all thigh muscles, including the sartorius, gracilis and pelvic muscles such as the gluteus muscles and iliacus muscle, whereas the semitendinosus muscle was relatively less involved.

## Discussion

In this study, we comprehensively analyzed the clinical and molecular data of 41 unrelated families with LGMD originating from Southeast China. Our study revealed that LGMD-R2-dysferlin-related and LGMD-R1-calpain3-related were the most common subtypes and that dystroglycanopathies were the most common childhood-onset subtypes. We reported five novel variants in LGMD-related genes, a rare subtype of LGMD-R18-*TRAPPC11*-related, the frequency of cardiac dysfunction and respiratory involvement and the features of muscle MRI findings in relevant subtypes of LGMDs.

Consistent with previous reports from China, Korea and Japan, the most frequent LGMD subtype identified was dysferlinopathy (approximately 36.3% in our families). This subtype is also frequent in Brazil, with a relative frequency of 31.3% [40, 41]. However, the frequency of dysferlinopathy is comparatively lower in some European populations, with a relative proportion of 10%–18.7% among LGMD patients [42]. The second most frequent subtype in our study was LGMD-R1-calpain3-related, accounting for 29.3% of the families, which partially differed from previous reports that LGMD-R1-calpain3-related is the most prevalent form in Eastern, Southern Europe and the US. In Italy, approximately 28.4% of LGMD patients had LGMD-R1-calpain3-related, and the frequency was similar to that in the Netherlands. A previous large-sample study in the US revealed that LGMD-R1-calpain3-related was the most frequently diagnosed subtype (17% of LGMD phenotypes), followed by dysferlinopathy (16%) and LGMD-R9-*FKRP*-related (9%). However, in contrast, the most frequent autosomal recessive LGMD was LGMD-R9-*FKRP*-related in Denmark and the United Kingdom. The reported frequency of sarcoglycanopathies varied among each region in China, with the highest frequency of sarcoglycanopathies (29.2%) in the South region. In other regions of China, the frequencies of sarcoglycanopathies ranged from 7.7 to 15.4%; however, sarcoglycanopathies were not found in our cohort or in Korean studies. In Europe, sarcoglycanopathies are often considered the second most frequent form, with prevalence rates of 15–18.1% in Italy and 27% in the Netherlands. In the Brazilian population, sarcoglycanopathies are the most frequent cause of severe autosomal recessive LGMD (68%) and childhood-onset subtypes[43].

In our cohort, dystroglycanopathies were the most common subtypes before 11 years of age. A single-center study from Taiwan revealed that dystroglycanopathies were identified among 23.1% of individuals with LGMD, the same percentage as that for dysferlinopathy, the most common diagnosis, accounting for 50% of subtypes with onset before 11 years of age [44]. Overall, the most common subtypes in China were LGMD-R2-dysferlin-related and LGMD-R1-calpain3-related; however, the frequency of dystroglycanopathies and sarcoglycanopathies varied greatly among different regions. This phenomenon could be due to the relatively small sample size of previous LGMD cohorts and the different prevalences of some LGMD subtypes among different geographic regions. In addition, the diagnosis of LGMD subtypes in the Northeast region of China was made only according to testing for some mutant defective proteins, and the diagnosis was not further confirmed by genetic screening; therefore, some LGMD subtypes could not be identified.

All of our patients with LGMD-R7-telethonin-related carried the homozygous mutation c.26_33dupAGGTGTCG, except for one with homozygous c.110 + 5G > A mutations. Interestingly, 14% of our families had LGMD-R7-telethonin-related, which has rarely been reported in other LGMD subtype cohorts worldwide. To date, a total of 30 patients with LGMD-R7-telethonin-related have been diagnosed in the Chinese population, and five different pathogenic *TCAP* mutations have been identified in these patients [45, 46]. A previous study in Brazil reported that all of the families with LGMD-R7-telethonin-related were homozygous for a c.157C > T(p.Gln53*) mutation in *TCAP*, which has also been detected in Caucasian and Portuguese populations but is not found in the Chinese population [47, 48]. In addition, the hotspot variants in Europe and South Asia are c.75G > A and c.32C > A, respectively. In China, half of patients with LGMD-R7-telethonin-related carried at least one allele c.26_33dupAGGTGTCG mutation in *TCAP,* and 36.7% of patients were homozygous for this mutation.

We analyzed genotype–phenotype correlations with age at onset and clinical severity of disease in those with LGMD-R1-calpain3-related and dysferlinopathy. Our study showed that compared with two missense mutations, one missense/null mutation in *CAPN3* resulted in relatively late onset, but only 4 patients had one missense/null mutation in our cohort, and this association should be investigated in larger patient populations in the future. Previous studies have revealed genotype–phenotype correlations with age at onset in those with LGMD-R1-calpain3-related and dysferlinopathy, with earlier symptom onset in patients with two null mutations than in patients with at least one missense mutation [49]. Differences in age at onset according to dysferlinopathy genotype were similar to the current results. Our data also revealed that patients with two null mutations tended to have earlier symptom onset than those with at least one missense mutation, although the difference was not statistically significant. Previous Japanese studies have found that the c.2997G > T mutation in the *DYSF* gene is associated with relatively late onset [50, 51]; however, this mutation was not found in our study. Our study also evaluated the effects of null mutations on the clinical severity of dysferlinopathy, but we did not find a significant difference between patients with two null mutations and those with at least one missense mutation. This finding might be because of different disease durations between the two groups. Additionally, intrafamilial variability was observed in dysferlinopathy, suggesting that other additional factors, such as environmental factors and modifier gene(s), also play important roles in the clinical phenotype of the disease. These data suggest that the clinical severity of dysferlinopathy is related to the type(s) of mutation(s), the presence of mutations in residual inner domains, and other unknown modifying factors.

Cardiac involvement is common in many subtypes of LGMDs, which could lead to an increased risk of sudden death [52]. A total of 22.0% (9/41) of all patients identified in our study had cardiac dysfunction. The ECG findings of our patients included conduction defects (right bundle branch block and intraventricular block), arrhythmias (atrial flutter and sinus arrhythmia), and early repolarization syndrome. Cardiac complications are the most common causes of death in *LMNA*-related muscular dystrophies and were found in only one patient with *LMNA*-related muscular dystrophy in this study [53]. This event corroborated previous reports that cardiac involvement in patients with *LMNA* mutations is more frequent and severe than in patients with other types of muscular dystrophies. Another patient with LGMD-R9-*FKRP*-related showed a right bundle branch block, which is not an uncommon finding in the population and is not directly related to the diagnosis of cardiomyopathy. However, her CMR indicated cardiomyopathy involving fatty infiltration of the subepicardial layer of the LV. Therefore, CMR can be used to detect early cardiac abnormalities in patients with LGMDs, monitor disease progression and assess treatment effects during follow-up. Furthermore, early diagnosis is necessary for the timely commencement of cardiac management, and therapeutic interventions are appropriate for patients with severe cardiac arrhythmias.

A total of 15.4% of all patients experienced restrictive respiratory insufficiency, but none required nocturnal noninvasive ventilation, which warrants longer follow-up. Previous studies revealed that 80% of patients with LGMD-R9-*FKRP*-related experienced respiratory impairment, and 3%–45% required assisted ventilation [54, 55]. One of two patients with LGMD-R9-*FKRP*-related in our study had relatively severe respiratory insufficiency and cardiac involvement. Our findings support that cardiorespiratory dysfunction is a frequent complication of LGMD-R9-*FKRP*-related. Respiratory impairment was not observed in our patients with other subtypes of dystroglycanopathies, suggesting that respiratory involvement could be more frequent in LGMD-R9-*FKRP*-related than in other dystroglycanopathy subtypes. In addition, we reported that a rare subtype, LGMD-R18-*TRAPPC11*-related, presented as severe respiratory impairment. Although no correlation between muscle weakness and respiratory insufficiency was observed in previous reports, patients with LGMD-R9-*FKRP*-related and LGMD-R18-*TRAPPC11*-related with respiratory involvement showed a relatively severe phenotype with walking aid dependency. This finding requires further investigation in larger numbers of patients with LGMD subtypes at greater risk for developing respiratory complications. It is important for clinicians to be aware of symptoms of ventilatory insufficiency in patients with LGMDs and to monitor pulmonary function during follow-up.

Age at onset was predominantly in childhood in patients with dystroglycanopathies, LGMD-R10-*TTN*-related and *LMNA*-related muscular dystrophy and in childhood or adolescence in those with LGMD-R1-calpain3-related, dysferlinopathy, LGMD-R7-telethonin-related and LGMD-R18-*TRAPPC11*-related. A loss of ambulation was observed in one patient with *IPSD*-related dystroglycanopathy at the early age of 21, suggesting that *IPSD*-related dystroglycanopathy phenotypes are relatively severe. Previous studies revealed that patients with LGMD-R18-*TRAPPC11*-related had infantile-onset or early childhood onset and presented with progressive muscle weakness and extramuscular manifestations, including motor delay, ataxia, cataracts, liver disease, and intellectual disability [56, 57]. Our patient with LGMD-R18-*TRAPPC11*-related had a relatively late onset of 19 years and presented with a myopathic syndrome and signs of foot drop associated with fatty liver and diabetes.

We performed muscle MRI imaging on 28 patients with different subtypes of LGMD. LGMD-R1-calpain3-related and LGMD-R2-dysferlin-related exhibited clinical similarity in some aspects; however, some features of muscle MRI patterns can help distinguish these two subtypes. The patients with LGMD-R1-calpain3-related showed more severe fatty infiltration of the posterior thigh muscles than those with LGMD-R2-dysferlin-related, consistent with the findings of a previous report. However, there were no differences in the fatty infiltration of the anterior thigh muscles between patients with LGMD-R1-calpain3-related and those with LGMD-R2-dysferlin-related, although fatty infiltration of these muscles was previously reported to be less severe in patients with LGMD-R1-calpain3-related [58]. Furthermore, muscle edema was more frequent and severe in the lower legs of patients with LGMD-R2-dysferlin-related than in patients with LGMD-R1-calpain3-related. The edema pattern in LGMD-R2-dysferlin-related was rather heterogeneous and prominent and involved different compartments of the lower legs. In contrast to patients with other subtypes, muscle imaging in our patient with LGMD-R9-*FKRP*-related indicated more severe fatty infiltration of the peroneus longus muscle and extensor digitorum longus, while the tibialis anterior muscle was relatively spared. According to previous literature, a specific pattern of muscle imaging in LGMD-R9-*FKRP*-related could facilitate differential diagnosis from other LGMDs [59]. In addition, muscle imaging in patients with LGMD-R18-*TRAPPC11*-related revealed an advanced stage pattern that has not been previously reported, and more patients must be enrolled for further analysis.

In conclusion, we described the detailed clinical phenotypes, muscle imaging findings, and genetic spectrum of an LGMD cohort of 50 patients in 41 families. We comprehensively analyzed the frequency of LGMD subtypes in different regions in China, which revealed that LGMD-R2-dysferlin-related and LGMD-R1-calpain3-related were the most common subtypes. We described the distinct characteristic patterns of muscle imaging in different subtypes of LGMDs, which may improve the differential diagnoses of the diseases. We reported a rare subtype of LGMD, recurrent mutations and novel pathogenic mutations, which contributed to expanding the clinical phenotype and genetic spectrum of LGMD. Cardiac involvement and respiratory insufficiency are common complications in many subtypes of LGMDs, and it is essential to regularly screen cardiac and pulmonary function in the future management of LGMDs. These results improve our understanding of the epidemiology of different LGMD subtypes in China, facilitating diagnostic processes, allowing for the timely provision of cardiorespiratory treatment and benefitting future clinical trials for LGMDs.

### Supplementary Information

**Additional file 1.** Clinical and molecular data of 81 patients suspected of LGMD.

**Additional file 2**: ** Figure S1**. Detailed variant interpretation of whole-exome sequencing (WES) and targeted next-generation sequencing (NGS).

**Additional file 3**: **Figure S2**. The mean score of all 12 muscles evaluated in the thigh and the tensor fasciae latae muscle in LGMD-R1-calpain3-related and LGMD-R2-dysferlin-related.

**Additional file 4**: **Figure S3**. The mean score of the 8 muscles evaluated in the lower legs in LGMD-R1-calpain3-related and LGMD-R2-dysferlin-related.

**Additional file 5.** Specific primers for Sanger sequencing of mutations in LGMD-related genes.

## Data Availability

The datasets supporting the conclusions of this article are included within the article (and its additional files).

## Abbreviations

LGMD: Limb-girdle muscular dystrophy
CK: Creatine kinase
IHC: Immunohistochemistry
WES: Whole-exome sequencing
NGS: Next-generation sequencing
NMD: Neuromuscular disease
GATK: Genome analysis toolkit
SNV: Single-nucleotide variant
ACMG: American College of Medical Genetics and Genomics
CAPN3: Calpain 3
DYSF: Dysferlin
TCAP: Titin cap
FKRP: Fukutin-related protein
TTN: Titin
POMT1: Protein-O-mannosyltransferase 1
LMNA: Lamin A/C
POMT2: Protein-O-mannosyltransferase 2
TRAPPC11: Trafficking protein particle complex 11
ISPD: Isoprenoid synthase domain containing
LV: Left ventricle
CMR: Cardiac magnetic resonance
FVC: Forced vital capacity
FEV1: Forced expiratory volume in 1 s
