# Supplementary material for: Clinical features, imaging findings and molecular data of limb-girdle muscular dystrophies in a cohort of Chinese patients
Source: Orphanet J Rare Dis. 2023 Nov 16;18:356. doi: 10.1186/s13023-023-02897-x (PMC10652577; doi:10.1186/s13023-023-02897-x)
Supplement: Supplementary file 1 — Additional file 1. Clinical and molecular data of 81 patients suspected of LGMD. [file 13023_2023_2897_MOESM1_ESM.docx]

| No | Gene  **Table 1** clinical and molecular data of 81 patients suspected of LGMD | Age/  Gender | Onset age | Distribution | | | | Hypertrophy | Contracture | Foot drop | Wing scapula | CK level | Cardiac dysfunction | Respiratory  Involvement | Mutations |
| --- | --- | --- | --- | --- | --- | --- | --- | --- | --- | --- | --- | --- | --- | --- | --- |
|  |  |  |  | **Neck**  **flexors** | **Upper**  **limbs** | **Lower**  **limbs** | **Distal** |  |  |  |  |  |  |  |  |
| 1 | LMNA | 37/M | 5 | + | + | + | + | - | + | + | + | 1099 | LV dysfunction | Not available | Hetero c.746G>A |
| 2 | CAPN3 | 25/M | 16 | - | + | + | - | - | - | - | - | 6598 | Normal | Normal | Homo c.1621C>T |
| 3 | CAPN3 | 48/M | 28 | - - | + | + | - | - | - | - | + | 2178 | Not available | Not available | Comp hetero  c.433C>T  c.1621C>T |
| 4 | CAPN3 | 43/M | 35 | - | + | + | - | - | - | - | + | 3269 | [Tachycardia](javascript:;)  LV dysfunction | Normal | Comp hetero  c.2050+1G>A c.2120A>G |
| 5 | CAPN3 | 40/F | 28 | - | + | + | - | - | - | - | - | 1638 | Not available | Not available | Comp hetero  c.2050+1G>A c.2120A>G |
| 6 | CAPN3 | 39/M | 30 | + | + | + | - | - | - | - | + | 1400 | Normal | Normal | Comp hetero  c.2050+1G>A c.2120A>G |
| 7 | CAPN3 | 34/M | 12 | + | + | + | + | + | - | - | + | 564 | Normal | Not available | Comp hetero  c.2305C>T  c.1343G>A |
| 8 | CAPN3 | 17/M | 17 | - | - | + | - | + | - | - | - | 7250 | Normal | Normal | Comp hetero  c.1720T>G  c.2306G>C |
| 9 | CAPN3 | 41/M | 20 | + | + | + | - | - | - | - | - | 446 | Tachycardia  LV dysfunction | Not available | Homo  c.2263G>A |
| 10 | CAPN3 | 38/F | 13 | + | + | + | + | - | - | - | + | 1357 | Not available | Not available | Homo  c.2263G>A |
| 11 | CAPN3 | 27/M | 24 | - | + | + | + | + | - | - | - | 5241 | Normal | Not available | Comp hetero  c.2120A>G c.1451T>C |
| 12 | CAPN3 | 24/F | 19 | - | + | + | - | - | - | - | - | 3501 | Normal | Normal | Comp hetero  c.1855C>T  c.77C>G |
| 13 | CAPN3 | 15/F | 12 | + | + | + | - | - | - | - | - | 1837 | Normal | Normal | Homo c.2306G>C |
| 14 | CAPN3 | 30/F | 22 | - | + | + | - | + | - | - | - | Not available | Not available | Not available | Homo c.1817C>T |
| 15 | CAPN3 | 47/M | 27 | - | + | + | - | - | - | - | + | 2123 | Not available | Not available | Comp hetero  c.2306G>C c.2120A>G |
| 16 | CAPN3 | 20/M | 10 | + | + | + | - | - | - | - | - | 2144 | Normal | Normal | Comp hetero  c.1621C>T  c.1693C>T |
| 17 | DYSF | 51/F | 21 | + | + | + | + | - | - | + | - | 1142 | Tachycardia | Respiratory insufficiency | Homo c.863A>T |
| 18 | DYSF | 60/F | 32 | + | + | + | + | - | + | - | - | 1579 | Not available | Not available | Homo c.863A>T |
| 19 | DYSF | 20/M | 19 | - | - | + | - | - | - | - | - | 31740 | Normal | Normal | Comp hetero  c.6217A>G c.5350C>T |
| 20 | DYSF | 35/M | 30 | + | + | + | - | - | - | - | - | 5283 | Normal | Not available | Comp hetero  c.927C>G  c.4700delT |
| 21 | DYSF | 54/M | 48 | - | + | + | - | - | - | - | - | 3170 | LV dysfunction | Normal | Comp hetero  c.965T>C  c.1667T>C |
| 22 | DYSF | 24/M | 18 | + | + | + | + | - | - | + | - | 10530 | Normal | Not available | Homo  c.1667T＞C |
| 23 | DYSF | 21/F | 20 | + | + | + | - | - | - | + | - | 6551 | Normal | Normal | Comp hetero  c.3102C>G  c.5836C>T |
| 24 | DYSF | 17/M | 13 | - | + | + | + | - | - | + | - | 4474 | Left ventricular high voltage | Not available | Comp hetero  c.3102C>G  c.5836C>T |
| 25 | DYSF | 33/F | 23 | - | - | + | - | + | - | - | - | 5407 | Not available | Not available | Homo  c.799_800delTT |
| 26 | DYSF | 34/M | Not yet | - | - | - | - | - | - | - | - | 9405 | Normal | Normal | Homo  c.799_800delTT |
| 27 | DYSF | 33/F | 30 | - | + | - | - | - | - | - | - | 3586 | Normal | Normal | Comp hetero  c.937+1G>A  c.3113G＞A |
| 28 | DYSF | 37/M | 17 | + | + | + | + | - | - | + | + | 1808 | Normal | Not available | Homo c.5903G>A |
| 29 | DYSF | 29/F | 26 | + | + | + | - | - | - | - | + | 19611 | Normal | Normal | Comp hetero  c.89-2A>G  c.2810+1G>A |
| 30 | DYSF | 29/F | 25 | - | - | + | - | - | - | - | - | 12140 | Normal | Normal | Comp hetero  c.4325delG c.5947-1G>A |
| 31 | DYSF | 40/M | 28 | - | - | + | + | - | - | + | - | 3284 | Normal | Respiratory insufficiency | Comp hetero  c.863A>T  55 exon deletion |
| 32 | DYSF | 23/M | 20 | - | - | - | + | - | - | - | - | 9255 | Normal | Normal | Comp hetero  c.712A>T  c.4200delC |
| 33 | DYSF | 33/F | 18 | - | - | + | + | - | - | - | - | 2987 | Normal | Normal | Homo c.1644delA |
| 34 | DYSF | 27/M | 26 | - | - | + | - | + | - | - | - | 4358 | Normal | Not available | Comp hetero  c.5694dupT  c.937+1G>A |
| 35 | TCAP | 25/F | 22 | - | - | + | + | - | + | + | - | 678 | Normal | Normal | Homo  c.26_33dupAGGTGTCG |
| 36 | TCAP | 33/F | 15 | - | - | + | + | - | - | - | - | 872 | Normal | Normal | Homo  c.26_33dupAGGTGTCG |
| 37 | TCAP | 33/M | 16 | - | - | + | + | - | - | - | - | 2361 | Not available | Not available | Homo  c.26_33dupAGGTGTCG |
| 38 | TCAP | 48/M | 17 | + | + | + | + | - | - | - | - | 408 | Normal | Not available | Homo  c.110+5G>A |
| 39 | TCAP | 32/M | 22 | + | + | + | - | + | -- | - | + | 974 | Not available | Normal | Homo  c.26_33dupAGGTGTCG |
| 40 | TCAP | 41/F | 31 | + | + | + | + | + | - | - | - | 1357 | Normal | Normal | Homo  c.26_33dupAGGTGTCG |
| 41 | TCAP | 37/F | 35 | - | - | + | + | - | - | - | - | 823 | Normal | Not available | Homo  c.26_33dupAGGTGTCG |
| 42 | FKRP | 29/F | 10 | + | + | + | + | - | - | + | + | 1325 | Cardiomyopathy | Respiratory insufficiency | Comp hetero  c.948delC c.545A>G |
| 43 | FKRP | 8/F | 6 | + | + | + | - | + | - | -- | - | 6300 | Normal | Normal | Comp hetero  c.545A>G c.206_208delCCT |
| 44 | TTN | 23/M | 8 | - | - | + | - | - | - | - | - | 1025 | Normal | Not available | Homo  c.7509T>C |
| 45 | TTN | 25/F | 11 | - | + | + | + | + | - | - | - | 875 | Normal | Not available | Homo  c.7509T>C |
| 46 | POMT1 | 15/M | 5 | - | + | + | - | + | - | - | - | 1895 | Early repolarization syndrome | Not available | Comp hetero  c.2210_2221del  c.2164G>A |
| 47 | POMT1 | 25/F | 10 | - | + | + | - | - | - | - | - | 1038 | Normal | Not available | Comp hetero  c.2210_2221del  c.2164G>A |
| 48 | POMT2 | 10/F | 4 | + | + | + | - | + | - | - | - | 12671 | Normal | Not available | Comp hetero  c.511C>T  c.365G>T |
| 49 | TRAPPC | 33/F | 19 | + | + | + | + | - | - | + | - | 420 | Normal | Respiratory insufficiency | Homo  c.2938G>A |
| 50 | ISPD | 24/M | 4 | + | + | + | + | + | - | - | - | 2807 | Tachycardia | Normal | Homo  c.1114_1116del |
| 51 | GNE | 31/M | 23 | + | + | + | + | - | - | + | - | 533 | Normal | Respiratory insufficiency | Comp hetero  c.830G>A  c.1664C>T |
| 52 | GNE | 29/M | 27 | + | - | + | + | - | - | - | - | 1162 | Normal | Respiratory insufficiency | Homo  c.830G>A |
| 53 | GNE | 35/F | 21 | - | - | + | + |  |  |  |  | 893 | Normal | Normal | Homo  c.830G>A |
| 54 | PYGM | 18/F | 8 | + | + | + | - | - | - | - | - | 1148 | [Valvular](javascript:;) [heart](javascript:;)  [disease](javascript:;) | Not available | Comp hetero  c.1643C>A  c.475G>A |
| 55 | PYGM | 15/M | 7 | + | + | + | - | - | - | - | - | 942 | Not available | Not available | Comp hetero  c.1643C>A  c.475G>A |
| 56 | HNRNPA1 | 52/M | 51 | + | + | + | + | - | - | + | - | 1821 | Normal | Normal | Hetero  c.940G>A |
| 57 | HNRNPA1 | 58/M | 48 | + | + | + | + | - | - | - | - | 795 | Not available | Not available | Hetero  c.940G>A |
| 58 | HNRNPA1 | 63/F | 56 | + | + | + | - | - | - | - | - | 578 | Bradycardia | Not available | Hetero  c.940G>A |
| 60 | HNRNPA1 | 71/M | 55 | + | + | + | + | - | - | + | + | 863 | Not available | Normal | Hetero  c.940G>A |
| 61 | PNPLA2 | 41/M | 34 | + | + | + | - | - | - | - | - | 473 | Bradycardia | Not available | Homo  c.757+1G>T |
| 62 | Uncertain | 37/M | 32 | + | + | + | - | - | - | + | - | 1600 | Normal | Normal | MYH7 Hetero  c.1322C>T  TRIM32 Hetero  c.409C>T |
| 63 | Uncertain | 48/F | 43 | + | + | + | + | - | - | - | - | 1442 | [Intraventricular](javascript:;)  [block](javascript:;) | Respiratory insufficiency | DYSF Hetero  c.1170C>G  c.6057-43C>T |
| 64 | Uncertain | 41/M | 37 | + | + | + | - | - | - | - | - | 999 | Normal | Normal | COL6A1 Hetero  c.859-8T>C  LAMA2 Hetero  c.2794A>C |
| 65 | Uncertain | 23/F | 21 | + | + | + | - | + | - | - | - | 842 | Normal | Normal | PLEC Hetero  c.115A>G  TTN Hetero c.96661G>A |
| 66 | Uncertain | 37/M | 33 | - | + | + | - | - | - | - | + | 1244 | [Arrhythmia](javascript:;) | Not available | / |
| 67 | Uncertain | 45/M | 20 | - | + | + | - | - | - | - | - | 465 | Normal | Respiratory insufficiency | TTN Hetero  c.39494G>A |
| 68 | Uncertain | 47/M | 45 | - | - | + | - | - | - | - | - | 752 | Tachycardia | Normal | LAMA2 Hetero  c.470C>G |
| 69 | Uncertain | 58/M | 51 | + | + | + | + | - | - | - | - | 825 | Normal | Not available | COL6A1 Hetero  c.694A>T |
| 70 | Uncertain | 31/M | 29 | + | - | + | - | + | - | - | - | 293 | Normal | Normal | PLEC Hetero  c.2516T>C  c.6796_6797delinsTT |
| 71 | Uncertain | 50/M | 44 | + | + | + | + | - | - | - | + | 6385 | Arrhythmia | Respiratory insufficiency | / |
| 72 | Uncertain | 42/M | 41 | + | - | + | - | - | - | - | - | 7000 | Multifocal ventricular premature beat | Not available | TTN Hetero  c.36654G>T |
| 73 | Uncertain | 20/M | 17 | + | + | + | - | + | - | - | + | 4664 | Normal | Normal | RYR1 Hetero  c.8305G>A  LDB3 Hetero c.415G>A |
| 74 | Uncertain | 43/F | 35 | + | - | + | + | - | - | - | + | 3179 | Normal | Not available | / |
| 75 | Uncertain | 18/F | 15 | - | - | + | + | - | - | + | - | 1600 | Normal | Normal | TTN Hetero  c.36654G>T  c.38726-1G>A |
| 76 | Uncertain | 27/F | 24 | - | - | + | - | - | - | - | - | 850 | Normal | Normal | / |
| 77 | Uncertain | 37/F | 30 | + | + | + | - | - | - | - | - | 1831 | Normal | Not available | CAPN3 Hetero  c.593A>G  COL6A3 Hetero  c.4697C>T |
| 78 | Uncertain | 45/M | 41 | - | - | + | - | - | - | - | - | 327 | Normal | Normal | / |
| 79 | Uncertain | 28/M | 12 | + | + | + | + | - | + | - | + | 893 | Not available | Not available | NEB Hetero  c.19550G>A |
| 80 | Uncertain | 43/M | 18 | + | + | + | - | - | - | - | + | 578 | Normal | Not available | COL6A3 Hetero  c.958G>A  TTN Hetero  c.99976+4A>G |
| 81 | Uncertain | 45/M | 20 | + | + | + | - | - | - |  | + | 1005 | Tachycardia | Not available | / |

LGMD, limb-girdle muscular dystrophy; M, male; F, female; CK, creatine kinase; +, involved; -, not involved; /, uncertain.
