# Supplementary figures and images for: Clinical features, imaging findings and molecular data of limb-girdle muscular dystrophies in a cohort of Chinese patients
Source: Orphanet J Rare Dis. 2023 Nov 16;18:356. doi: 10.1186/s13023-023-02897-x (PMC10652577; doi:10.1186/s13023-023-02897-x)

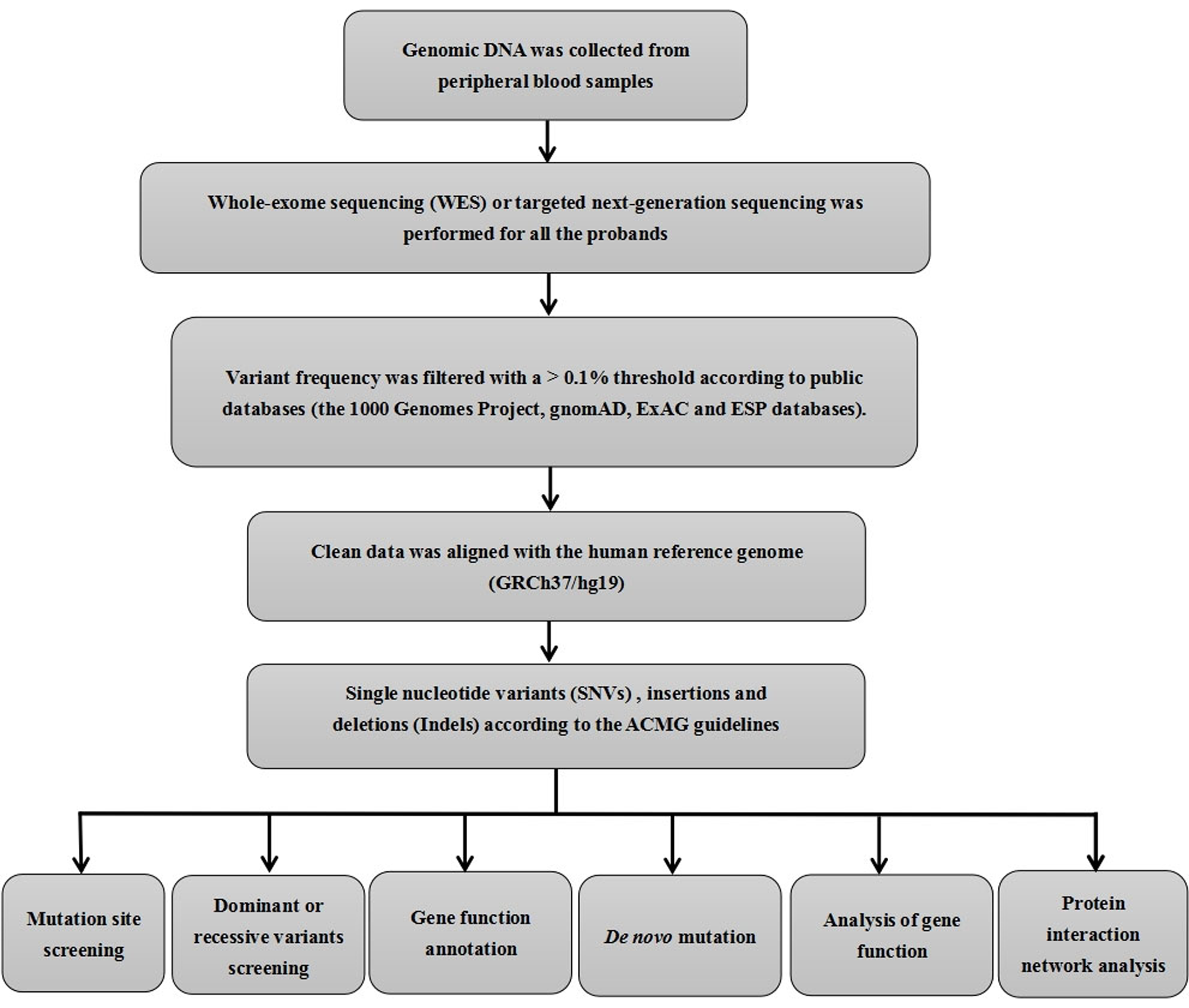

Supplement: Supplementary file 2 — Additional file 2: Figure S1. Detailed variant interpretation of whole-exome sequencing (WES) and targeted next-generation sequencing (NGS). [file 13023_2023_2897_MOESM2_ESM.tif]

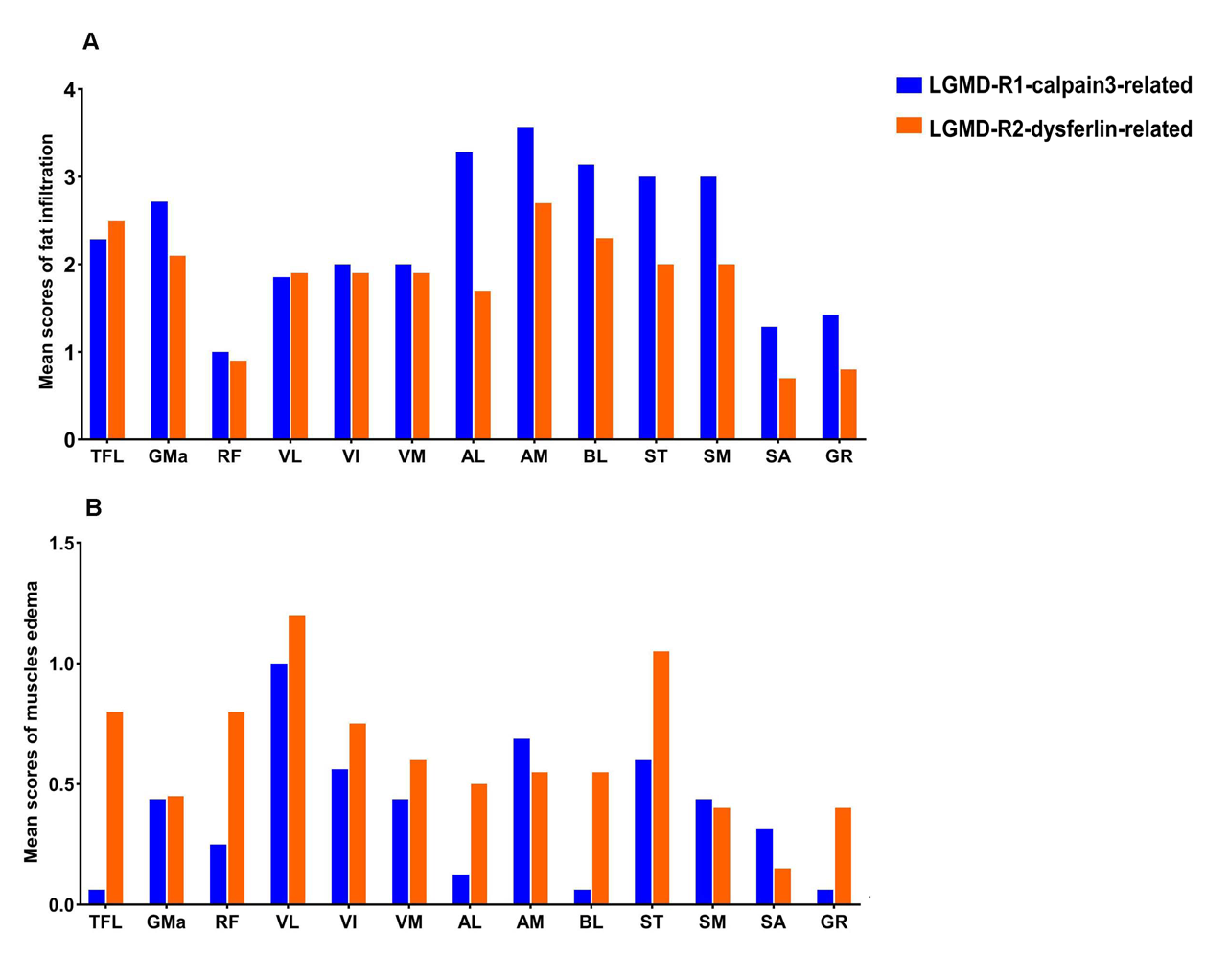

Supplement: Supplementary file 3 — Additional file 3: Figure S2. The mean score of all 12 muscles evaluated in the thigh and the tensor fasciae latae muscle in LGMD-R1-calpain3-related and LGMD-R2-dysferlin-related. [file 13023_2023_2897_MOESM3_ESM.tif]

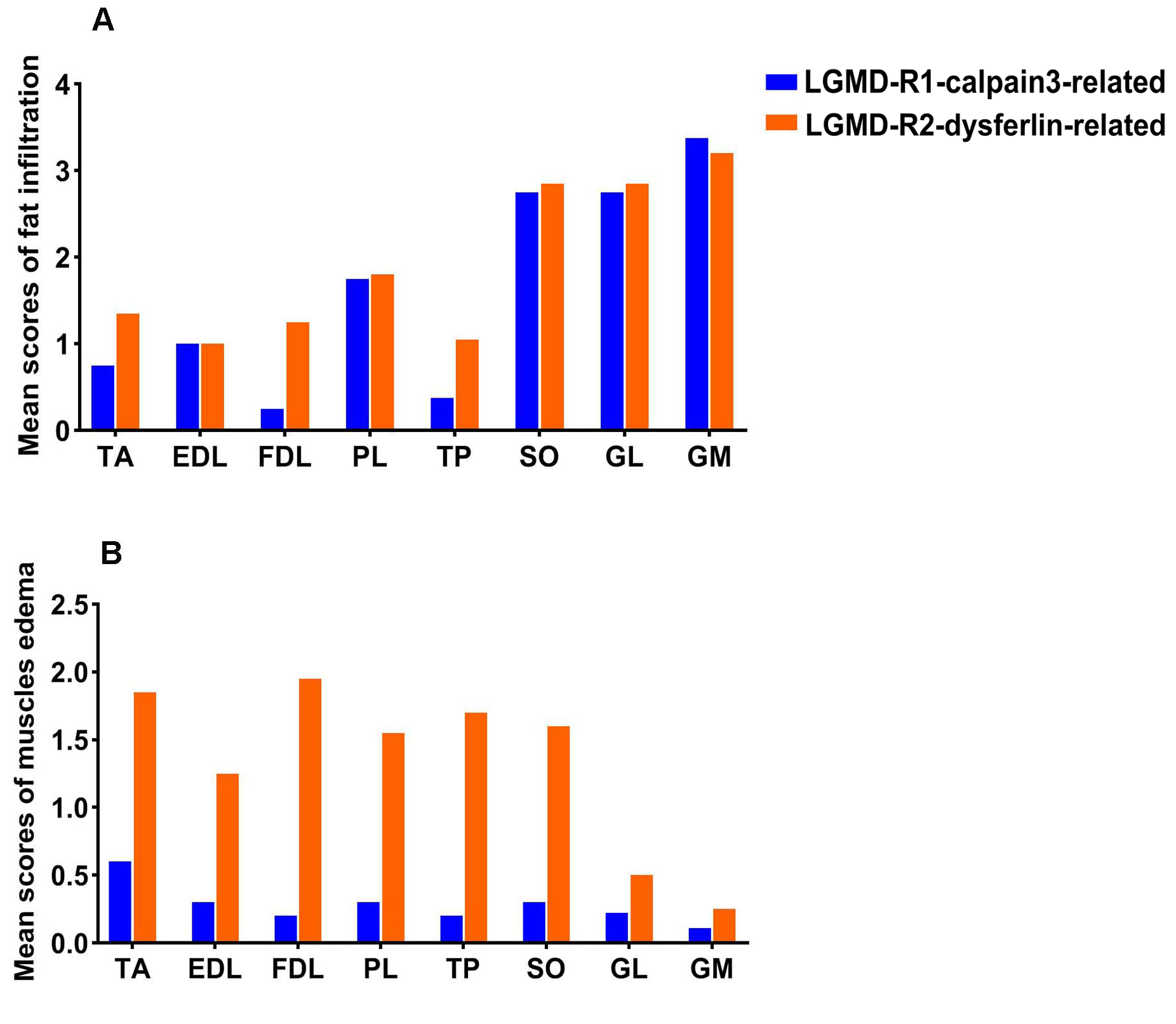

Supplement: Supplementary file 4 — Additional file 4: Figure S3. The mean score of the 8 muscles evaluated in the lower legs in LGMD-R1-calpain3-related and LGMD-R2-dysferlin-related. [file 13023_2023_2897_MOESM4_ESM.tif]
