# Supplementary material for: Clinical features, imaging findings and molecular data of limb-girdle muscular dystrophies in a cohort of Chinese patients
Source: Orphanet J Rare Dis. 2023 Nov 16;18:356. doi: 10.1186/s13023-023-02897-x (PMC10652577; doi:10.1186/s13023-023-02897-x)
Supplement: Supplementary file 5 — Additional file 5. Specific primers for Sanger sequencing of mutations in LGMD-related genes. [file 13023_2023_2897_MOESM5_ESM.docx]

**Table S1.** Specific primers for Sanger sequencing of mutations in *LMNA*, *CAPN3*, *DYSF*, *TCAP*, *FKRP*, *TTN*, *POMT1*, *POMT2*, *TRAPPC*11 and *ISPD*.

| **Name** | **Sequence (5'→3')** | **Target mutation** | **Location** |
| --- | --- | --- | --- |
| *LMNA*-E4-F | GGTAGTGGCTCATGGAGTAGG | c.746G>A | Exon 4 |
| *LMNA*-E4-F | CACAGGCCTGAGTTGGGCATCAC |  |  |
| *CAPN3*-E1-F | GCAGTTGCTTCCTTTCCTTGAAG | c.77C>G | Exon 1 |
| CAPN3-E1-R | CGGAGGTCTCTTCCAGACGAAC |  |  |
| *CAPN3*-E3-F | AAATGTCTAGTCACAAGGGAGTAAGTTACC | c.433C>T | Exon 3 |
| *CAPN3*-E3-R | CCCACAGTAAACAGGATGTCTTCC |  |  |
| *CAPN3*-E10-F | CTCAGAGAATAAGGCAAGCC | c.1343G>A | Exon 10 |
| *CAPN3*-E10-R | ACCATGTCACCACTAGCACC |  |  |
| *CAPN3*-E11-F | CTCTGAGAGGCAGCTGTGAATG | c.1451T>C | Exon 11 |
| *CAPN3*-E11-R | CACAAGTGGTCCTGATACCAGGAGC |  |  |
| *CAPN3*-E13-F | GGTTGAAACTGTGACATGGGTGAC | c.1621C>T | Exon 13 |
| *CAPN3*-E13-R | GAAGGACCCTGGAGGAGACG |  |  |
| *CAPN3*-E13-F | AGGACAGGATGTTCCTCCCG | c.1693C>T | Exon 13 |
| *CAPN3*-E13-R | CTCCATATCACCTCTCCAAATCTAACATGC |  |  |
| *CAPN3*-E13-F | AGGACAGGATGTTCCTCCCG | c.1720T>G | Exon 13 |
| *CAPN3*-E13-R | CTCCATATCACCTCTCCAAATCTAACATGC |  |  |
| *CAPN3*-E16-F | GTCAGTGGCAGAGATAGAGCTTG | c.1817C>T | Exon 16 |
| *CAPN3*-E16-R | CTGGGATATTCACCCAAGCAGCA |  |  |
| *CAPN3*-E16-F | GTCAGTGGCAGAGATAGAGCTTG | c.1855C>T | Exon 16 |
| *CAPN3*-E16-R | CTGGGATATTCACCCAAGCAGCA |  |  |
| *CAPN3*-E18-F | CCATGACATAATAGCACCGACAGG | c.2050+1G>A | Exon 18 |
| *CAPN3*-E18-R | CCAAGATGTTCTGAATCAGGGTCTC |  |  |
| *CAPN3*-E20-F | CTCCTGGGTTACAGAGTAGGCG | c.2120A>G | Exon 20 |
| *CAPN3*-E20-R | CTGCATTTCGCATCTCGTAGCTG |  |  |
| *CAPN3*-E21-F | GGAGATTCAGTGTGTGACCTCCATC | c.2263G>A | Exon 21 |
| *CAPN3*-E21-R | GAGGTCATTGGCCCTTGTTCAGCC | c.2263G>A | Exon 21 |
| *CAPN3*-E22-F | GGAGATTCAGTGTGTGACCTCCATC | c.2305C>T | Exon 22 |
| *CAPN3*-E22-R | GAGGTCATTGGCCCTTGTTCAGCC |  |  |
| *CAPN3*-E22-F | GGAGATTCAGTGTGTGACCTCCATC | c.2306G>C | Exon 22 |
| *CAPN3*-E22-R | GAGGTCATTGGCCCTTGTTCAGCC |  |  |
| *DYSF*-E2-F | GCATGTTTCTGATGTCCCAGAGC | c.89-2A>G | Exon 2 |
| *DYSF*-E2-R | CCTCAAGGACACTGCTGAGAAGG |  |  |
| *DYSF*-E7-F | GTTTAATGTGGGTAGCCGACGAGGGG | c.712A>T | Exon 7 |
| *DYSF*-E7-R | CAGAAAATGTGGATTGGGCACAG |  |  |
| *DYSF*-E8-F | CTTGTCCAATCCCCAGAACTGTG | c.799_800delTT | Exon 8 |
| *DYSF*-E8-R | GACAGGCACACACACGCATTC |  |  |
| *DYSF*-E9-F | GTGAGGGCTAAACACTGCTTAGAAC | c.863A>T | Exon 9 |
| *DYSF*-E9-R | GAGATGCTGTGCAGAGGGCTTG |  |  |
| *DYSF*-E10-F | GACTGCCTGTGTTTCCAAATGTTC | c.927C>G | Exon 10 |
| *DYSF*-E10-R | GGAGACACAGGTGAGTGTTACACC |  |  |
| *DYSF*-E10-F | GACTGCCTGTGTTTCCAAATGTTC | c.937+1G>A | Exon 10 |
| *DYSF*-E10-R | GGAGACACAGGTGAGTGTTACACC |  |  |
| *DYSF*-E11-F | GAATCTGAGTCCTGAGCTCATGGC | c.965T>C | Exon 11 |
| *DYSF*-E11-R | CCTTCCAGAGGGATGTGCAAT |  |  |
| *DYSF*-E19-F | CTGAGTTGGCATCTGGCACTGTCC | c.1644delA | Exon 19 |
| *DYSF*-E19-R | GCAGGGCAAGTGTTGATTTATTCCC |  |  |
| *DYSF*-E19-F | CTGAGTTGGCATCTGGCACTGTCC | c.1667T>C | Exon 19 |
| *DYSF*-E19-R | GCAGGGCAAGTGTTGATTTATTCCC |  |  |
| *DYSF*-E26-F | CTACCTGGAGCTGCCTTGGCCC | c.2810+1G>A | Exon 26 |
| *DYSF*-E26-R | GAAAGGACTGCTGCCTGGCAA |  |  |
| *DYSF*-E29-F | GCTCTCAAGCCATGCTGGTG | c.3102C>G | Exon 29 |
| *DYSF*-E29-R | GGTGTCTGTGCATGCTGTGTGC |  |  |
| *DYSF*-E39-F | CTCTTTGGGCTTGACCTGGAGAC | c.4200delC | Exon 39 |
| *DYSF*-E39-R | CCACTTCTCATTCAGGTCTCCGTAAG |  |  |
| *DYSF*-E39-F | CTCTTTGGGCTTGACCTGGAGAC | c.4325delG | Exon 39 |
| *DYSF*-E39-R | CCACTTCTCATTCAGGTCTCCGTAAG | c.4325delG | Exon 39 |
| *DYSF*-E43-F | AAAAGGAGGAGGAAGCAAAAGCAGAAAC | c.4700delT | Exon 43 |
| *DYSF*-E43-R | CTATGCTTTTGGCCCCTCTTAAAACAC |  |  |
| *DYSF*-E48-F | GTGGTCACCTCTGCGGTTGACC | c.5350C>T | Exon 48 |
| *DYSF*-E48-R | CGAGAACTGTATTCCATGGCCCAA |  |  |
| *DYSF*-E51-F | GTCTTCCCACAGGACCTGGCTC | c.5694dupT | Exon 51 |
| *DYSF*-E51-R | GATCACTCCTCCTGCTAGGCTTCC |  |  |
| *DYSF*-E52-F | GGCTGGCAGTGATCGAGAAAC | c.5836C>T | Exon 52 |
| *DYSF*-E52-R | CAGAGGGAACCCAGGACACAGG |  |  |
| *DYSF*-E52-F | GGCTGGCAGTGATCGAGAAAC | c.5947-1G>A | Exon 52 |
| *DYSF*-E52-R | CAGAGGGAACCCAGGACACAGG |  |  |
| *DYSF*-E52-F | GGCTGGCAGTGATCGAGAAAC | c.5903G>A | Exon 52 |
| *DYSF*-E52-R | CAGAGGGAACCCAGGACACAGG |  |  |
| *TCAP*-E1-F | ACTTATAGCATCTGACACCAGAGGG | c.26_33dupAGGTGTCG | Exon 1 |
| *TCAP*-E1-R | GCCCTGGAGAAATTTCTGGGGG |  |  |
| *TCAP*-E1-F | ACTTATAGCATCTGACACCAGAGGG | c.110+5G>A | Exon 1 |
| *TCAP*-E1-R | GCCCTGGAGAAATTTCTGGGGG |  |  |
| *FKRP*-E4-F | TTCTATGTCTCGTGGCTGCAG | c.948delC | Exon 4 |
| *FKRP*-E4-R | ACATCATCCCATGGGACTACGA |  |  |
| *FKRP*-E4-F | TTCTATGTCTCGTGGCTGCAG | c.545A>G | Exon 4 |
| *FKRP*-E4-R | ACATCATCCCATGGGACTACGA |  |  |
| *FKRP*-E4-F | TTCTATGTCTCGTGGCTGCAG | c.206_208delCCT | Exon 4 |
| *FKRP*-E4-R | ACATCATCCCATGGGACTACGA |  |  |
| *TTN*-E186-F | CAGACATGTGGTCACTTGGAACACT | c.75019T>C | Exon 186 |
| *TTN*-E186-R | TTCTGAGATCGAATTGCACCACCAC |  |  |
| *POMT1*-E20-F | CTCCAGAGGAGCATCTTCAG | c.2210-2221del | Exon 20 |
| *POMT1*-E20-R | CTAGTGTTTTCGGATCAAGA |  |  |
| *POMT1*-E20-F | TGACCGTGTGGACAGCAG | c.2164G>A | Exon 20 |
| *POMT1*-E20-R | CGGGATGAGGTTGAAGGGTCTT | c.2164G>A | Exon 20 |
| *POMT2*-E3-F | GACAGTTGTCAAGGGAACCCAAAG | c.365G>T | Exon 3 |
| *POMT2*-E3-R | AGAGGAGGCTGCAAATAATACAGAGA |  |  |
| *POMT2*-E5-F | GCTTCTTGTCATTGGGCTTTGATG | c.551C>T | Exon 5 |
| *POMT2*-E5-R | CAGCTTCAAGACAAGTCTCTGGG |  |  |
| *TRAPPC11*-E26-F | CAGTGGACTTTGTGACCTTGAAAAAGTC | c.2938G>A | Exon 26 |
| *TRAPPC11*-E26-R | GGTTTGTGTTCATATCCACATGACTTCT |  |  |
| *ISPD*-E8-F | GAAGGCACTTAAGGATATGAACAAATGTGG | c.1114_1116del | Exon 8 |
| *ISPD*-E8-R | GGCATTCAGTCATAGAAGTCATAGCTAGAG |  |  |

E = Exon; F = Forward; R = Reverse.
